# Combining whole-cell patch clamp and dye loading in acute brain slices with bulk RNA sequencing in embryonic to aged mice

**DOI:** 10.1016/j.xpro.2021.100439

**Published:** 2021-04-09

**Authors:** Yasmine Kamen, Ragnhildur Thóra Káradóttir

**Affiliations:** 1 Wellcome – Medical Research Council Cambridge Stem Cell Institute and Department of Veterinary Medicine, University of Cambridge, Cambridge CB2 0AW, United Kingdom; 2 Department of Physiology, Biomedical Centre, Faculty of Medicine, University of Iceland, Reykjavik, Iceland

**Keywords:** Cell isolation, RNA-seq, Microscopy, Molecular Biology, Neuroscience, Stem Cells

## Abstract

Single-cell electrophysiological recordings combined with dye loading and immunohistochemistry provide unparalleled single-cell resolution of cell physiology, morphology, location, and protein expression. When correlated with bulk RNA sequencing, these data can define cell identity and function. Here, we describe a protocol to prepare acute brain slices from embryonic and postnatal mice for whole-cell patch clamp, dye loading and post-hoc immunohistochemistry, and cell isolation for bulk RNA sequencing. While we focus on oligodendrocyte precursor cells, this protocol is applicable to other brain cells.

For complete details on the use and execution of this protocol, please refer to Spitzer et al. (2019).

## Highlights

• Preparation of acute brain slices from embryonic, young postnatal, and aged mice
• Whole-cell patch clamp, dye filling, and post-hoc labeling of glia in acute brain slices
• Isolating and bulk RNA sequencing cells that are homogeneous within conditions
• Combining physiology, morphology, location, and transcriptome to determine cell function

## Graphical abstract

**Figure:**
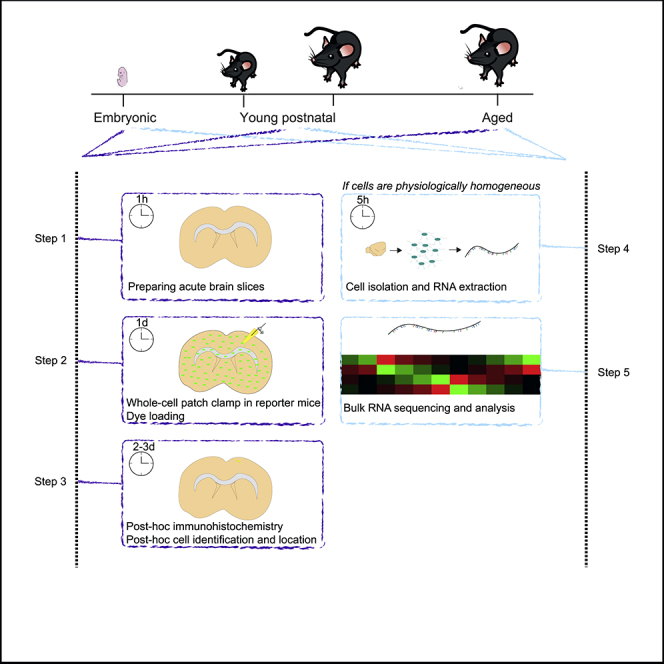

## Before you begin

Whole-cell patch clamp is typically used to study neuronal synaptic transmission and firing rate to understand neuronal signaling. However, the bioelectrical properties of cells can regulate cell biology beyond neuronal signaling. For instance, membrane potential is thought to regulate cell cycle progression in proliferative cells (Binggeli and Weinstein, 1986; Urrego et al., 2014). Further, neuronal activity regulates both neural stem cell (NSC) and oligodendrocyte precursor cell (OPC) proliferation and differentiation (Káradóttir and Kuo, 2018). Thus, studying the bioelectrical properties of a cell can contribute to our understanding of cell function in glia and progenitors. Combining standard whole-cell patch clamp with dye loading, as we describe here, allows for further morphological characterization of cells, and post-hoc immunohistochemistry can provide information on cell location and protein expression. In addition, these data can inform transcriptomic studies. In particular, if a cell population is found to be physiologically homogeneous within a condition, bulk RNA sequencing can become a simpler, faster, and more economical alternative to patch-seq (single-cell sequencing of RNA extracted following whole-cell patch clamp) or complement patch-seq data (as it provides deeper sequencing, and therefore better genome coverage) to characterize cell transcriptome and correlate bioelectrical properties with cell function.

Here, we focus on OPCs, which are equally distributed in the brain throughout life, but have altered proliferation and differentiation potential, physiology, and transcriptome with age (Dawson et al., 2003; Spitzer et al., 2019; Young et al., 2013). Thus, we describe a protocol to prepare acute brain slices in embryonic, neonatal, and adult mice for whole-cell patch clamp and dye loading, perform post-hoc immunohistochemistry on patched cells, and isolate cells for bulk RNA sequencing. We believe that this protocol is applicable to a range of non-neuronal cells, and have previously used it to record and stain astrocytes and microglia. Of note, we do not detail the electrophysiological technique or set up, but rather, focus on its application to OPCs. For further explanations on whole-cell patch clamp, we encourage the reader to consult these reviews and protocols (Gibb and Edwards, 1994; Hamill et al., 1981; Kornreich, 2007; Molleman, 2003; Sakmann and Neher, 1984, 1995; Sontheimer, 1995; Verkhratsky and Parpura, 2014).

### Prepare stock solutions for internal and external patch clamp and dye filling solutions

**Timing: 2 h, months to days before the recording day**

Here, we describe the preparation and storage of stock solutions that we use to make artificial cerebrospinal fluid (aCSF) or internal solution. These solutions can be made months to days in advance, as required, as the amounts described are sufficient for several months of daily patch clamp experiments.

1. Prepare a 500 mL stock of 1M NaOH in double-distilled water (ddH_2_O) by dissolving 20 g of NaOH in ddH_2_O. We use this solution to pH the 1× HEPES-buffered recording aCSF (step 43). 1M NaOH solution can be kept at room temperature (19°C–23°C). Discard the solution if any precipitate is observed.

**CRITICAL:** NaOH is corrosive. Handle with care and wear gloves.

2. Prepare a 500 mL stock of 1M NaH_2_PO_4_ in ddH_2_O (dissolve 68.995 g of NaH_2_PO_4_ in ddH_2_O). We use this stock solution to make up 10× bicarbonate- or HEPES-buffered aCSF (steps 23 and 24). 500 mL of 1M NaH_2_PO_4_ are sufficient for approximately one year of patch clamp experiments (depending on experiment frequency). This solution can be kept at room temperature (19°C–23°C), but should be discarded if any precipitate is observed. Keeping it in the fridge will minimize the risk of bacterial growth.
3. Prepare a 500 mL stock of 1M KCl in ddH_2_O (dissolve 27.275 g of KCl in ddH_2_O). We use this solution to make up 10× bicarbonate- or HEPES-buffered aCSF (steps 23 and 24). 1M KCl can be kept at room temperature (19°C–23°C), but should be discarded if any precipitate is observed. 500 mL of 1M KCl are sufficient for approximately one year of patch clamp experiments (depending on use).
4. Prepare a 10 mL stock of 4M NaCl in ddH_2_O (dissolve 2.338 g of NaCl in ddH_2_O). While this solution can be kept at room temperature (19°C–23°C), we recommend keeping it at 4°C, as it will be used to make up the internal solution (step 17), which has temperature sensitive components. Discard the solution if any precipitate forms.
5. Prepare a 10 mL stock of 1M HEPES in ddH_2_O (dissolve 2.383 g of HEPES in ddH_2_O). Keep this solution at 4°C. 10 mL are sufficient to make up several internal solutions (step 17). However, discard immediately if any growth is observed.
6. Prepare a 20 mL stock of 2M KOH in ddH_2_O (dissolve 2.244 g of KOH in ddH_2_O). While this solution can be kept at room temperature (19°C–23°C), we recommend keeping it at 4°C as it will be used to pH a K-gluconate based internal solution (step 19), which has temperature sensitive components. Discard the solution if any precipitate forms.
7. Prepare a 10 mL stock of 1M K-gluconate in ddH_2_O (dissolve 2.343 g of K-gluconate in ddH_2_O). We use K-gluconate to make up internal solution (step 17). Keep this solution at 4°C. We prepare 10 mL of stock solution, which can be used to prepare three internal solutions, as 1M K-gluconate can be kept for up to a year.
8. Prepare a 20 mL stock of 2M CsOH in ddH_2_O (dissolve 6.717 g of CsOH in ddH_2_O). While this solution can be kept at room temperature (19°C–23°C), we recommend keeping it at 4°C as it will be used to make up the internal solution (step 19), which has temperature sensitive components. Discard the solution if any precipitate forms.
9. Prepare a 20 mL stock of 2M of D-gluconic acid in ddH_2_O.

***Note:*** We purchase D-gluconic acid as a 51% solution in H_2_O (see key resources table). When calculating the molarity of the purchased solution, it is important to consider the density indicated by the supplier. Keep the 2M stock solution at 4°C, as it is used to make up internal solution.

10. Combine 2M CsOH and 2M D-gluconic acid (1:1) to make a 10 mL 1M Cs-gluconate stock. We use Cs-gluconate to prepare internal solution (step 17). Keep this stock solution at 4°C. We prepare 10 mL, as this is sufficient to make three internal solutions. Discard this solution after one year.

***Note:*** We use either K-gluconate or Cs-gluconate internal solutions, depending on the experiment. Thus, we typically have all the above stock solutions. However, if exclusively using a K-gluconate based internal solution, omit steps 8–10. Conversely, if only using a Cs-gluconate based solution, omit steps 6 and 7.

### Prepare drug aliquots for patch clamp

**Timing: 2 h, months to days before the recording day**

Here, we describe how to prepare and store stock solutions for various drugs we commonly use to record from OPCs. However, these are specific to our experiments, and may not always be needed. We keep these stock solutions for several months, depending on usage.

11. Prepare a 5 mL stock of 1M glycine in ddH_2_O (dissolve 0.375 g of glycine in ddH_2_O). We use glycine in our bath solution when recording NMDA receptor currents, as it is a receptor co-agonist. Prepare 0.1 mL aliquots (best for 1L of recording solution) and store at −20°C for up to one year. Avoid freeze-thaw.
12. Prepare a 3 mL stock of 50 mM of strychnine hydrochloride in ddH_2_O (dissolve 0.056 g of strychnine hydrochloride in ddH_2_O). We use strychnine in our bath solution when recording NMDA receptor currents, to block glycine receptors as we add glycine as an NMDA receptor co-agonist. Prepare 0.1 mL aliquots (for 1L of recording solution) and store at −20°C for up to one year. Avoid freeze-thaw.

**CRITICAL:** Strychnine hydrochloride is acutely toxic, and ingestion or inhalation can be fatal. When making up 50 mM strychnine hydrochloride stock solution from powder, wear gloves, a mask, and only handle in a fume hood until dissolved. We do not make up more than 3 mL at a time. Always wear gloves when handling solutions containing strychnine.

13. Prepare a 10 mL stock of 1M barium chloride in ddH_2_O (dissolve 2.443 g of barium chloride in ddH_2_O). We sometimes use barium chloride when we wish to block potassium conductance; this is particularly important when recording from glial cells in the adult gray matter. This solution does not need to be aliquoted, and should be kept at 4°C for up to one year.

**CRITICAL:** Barium chloride is toxic. When making up stock solution from powder, wear gloves, a mask, and only handle in a fume hood. Gloves should be worn when manipulating solutions containing barium.

14. Prepare a ∼1.56 mL stock of 30 mM kainate (we make up the entire 10 mg vial to avoid weighing out small quantities) in ddH_2_O. Prepare 0.1 mL aliquots (for 100 mL of 30 μM kainate, which is generally the amount we use for one day of recording) and store at −20°C until the day of use. Avoid freeze-thaw, and store for up to one year.
15. Prepare a ∼5.66 mL stock of 60 mM NMDA in ddH_2_O (we make up the entire 50 mg vial to avoid weighing out small quantities) in ddH_2_O. Prepare 0.1 mL aliquots (for 100 mL of 60 μM NMDA, which is generally the amount we use for one day of recording) and store at −20°C until the day of use. Avoid freeze-thaw, and store for up to one year.

### Prepare internal solution

**Timing: 1 day, months to days before the recording day**

16. Combine BAPTA, Mg_x_ATP and Na_x_GTP in 5 mL ddH_2_O.
17. Add Cs-gluconate (or K-gluconate), NaCl, CaCl_2_, and HEPES. See materials and equipment for the recipe.

**CRITICAL:** BAPTA will only fully dissolve when the solution is near pH 7.3. Use CsOH (or KOH if using a K-gluconate based internal) to bring the solution to pH 7.3.

18. Add Lucifer Yellow and allow it to dissolve or sonicate for a few seconds.
19. Measure pH and adjust to 7.2–7.4 with CsOH (or KOH if using a K-gluconate-based internal).

***Note:*** If the pH is higher than 7.4, the internal solution should be discarded and a new solution should be prepared. Do not use HCl to adjust pH, as this will alter the Cl^-^ concentration in the internal solution.

20. Add ddH_2_O to adjust to the final volume.
21. Measure osmolarity. Typically, osmolarity for this internal will be 290–300 mOsm.

***Note:*** If osmolarity does not fall between 290–310 mOsm, the solution should be discarded and a new solution should be prepared.

22. Aliquot the internal solution into 0.1 mL–0.5 mL aliquots (depending on how much is needed for one experimental day) and store at −20°C until the day of use. Discard aliquots after thawing them.

**CRITICAL:** While preparing the internal solution, maintain it on ice as much as possible, as ATP and GTP are temperature sensitive.

### Prepare 10× bicarbonate- and/or HEPES-buffered aCSF

**Timing: 20 min, up to a month before the recording day**

23. Prepare a 10× stock of bicarbonate-buffered aCSF for slicing, to keep the slices for 8–10 h, and for recordings (optional). Omit MgCl_2_, CaCl_2_, and glucose, which are added to the 1× solution, as different experiments may require different MgCl_2_ concentrations, CaCl_2_ precipitates easily, and glucose could promote bacterial growth if the solution is kept for more than a week.
  a. Add NaCl, NaHCO_3_, NaH_2_PO_4_ and KCl to ddH_2_O. See materials and equipment for the recipe.
24. Prepare a 10× stock of HEPES-buffered aCSF for recordings. Omit MgCl_2_, CaCl_2_, and glucose, as above.
  a. Add NaCl, HEPES, NaH_2_PO_4_ and KCl to ddH_2_O. See materials and equipment for the recipe.

***Note:*** If using a perfusion system where solutions do not continuously flow through (for instance, a gravity-fed perfusion with multiple drug lines), we recommend using a HEPES-buffered aCSF for recordings, as bicarbonate-buffered aCSF needs continuous bubbling with 95% O_2_ / 5% CO_2_ to maintain the pH between 7.2–7.4, and changes in solution pH will affect slice health and make results uninterpretable. If using bicarbonate-buffered aCSF for recordings, omit step 24.

***Note:*** 10× aCSF can be kept at 4°C for approximately one month. If precipitate is found in the solution, discard and prepare a new 10× stock.

### Prepare 5% agarose

**Timing: 10 min, months to days before the recording day**

**Figure 1:**
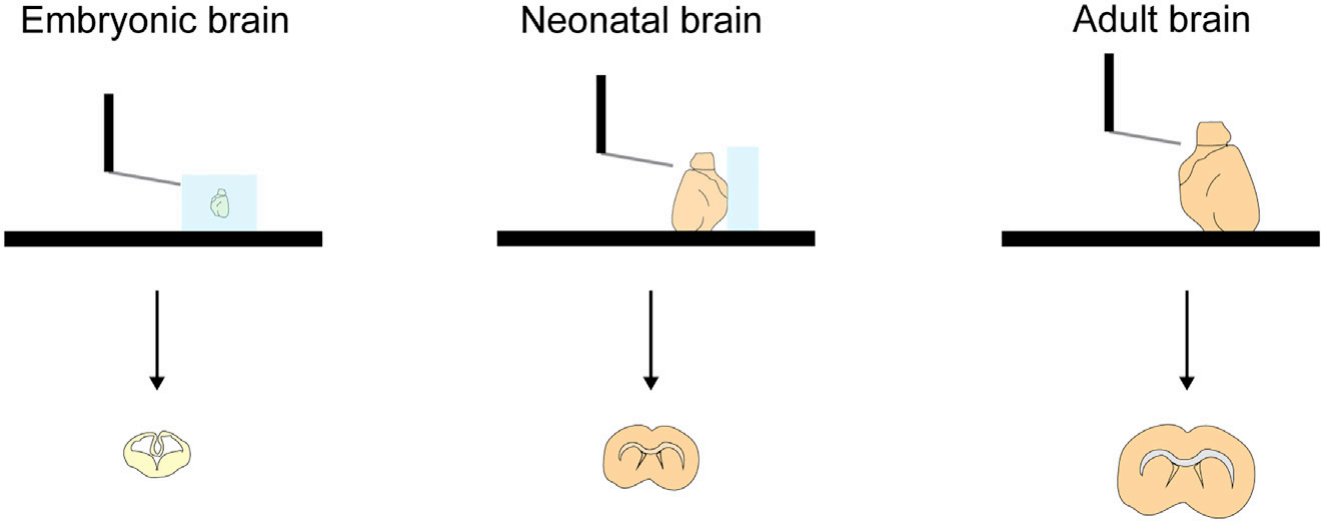
Preparing acute brain slices Diagram showing a side view of slicing through embryonic (left), neonatal (middle) and adult (right) brains to prepare coronal acute forebrain slices. For embryos, we dissect the brains and embed them in agarose before slicing through the block. To prepare neonatal slices, we suggest adding a block of agarose to support the brain. For adult slices, agarose is not necessary, as the brain is stiff enough to slice at low speed, but can be used if preferred.

We use 5% agarose when preparing slices from neonatal mice (up to P20) (Figure 1), to stabilize the brain, or in adult mice when the slicing angle requires a support to maintain the brain stable.

25. Dissolve low melting point agarose (2.5 g) in water (50 mL).
  a. Bring the agarose to boiling point using a hot plate.
26. Pour 50 mL of melted agarose into a 100 mm diameter petri dish (this should fill about three quarters of the height of the petri-dish).
27. Allow the agarose to set at 4°C overnight (10–20 h).

***Note:*** Keep the agarose in the fridge after it is made. Make sure to seal the agarose-containing petri dish with parafilm to prevent it from drying out. We recommend only making one or two dishes at the time, as they can get mouldy if kept for more than three months. Discard immediately if any growth is observed.

### Prepare 4% paraformaldehyde (PFA) aliquots

**Timing: 2 h, months to days before the recording day**

We typically prepare PFA in large quantities (1–2L) for transcardiac perfusions, and set aside approximately 250 mL to prepare aliquots to fix patched slices. Thus, if preparing PFA solely to fix patched slices, we recommend preparing 250 mL of solution. This should be sufficient for ∼50 patch clamp experiments.

28. Using a hot plate, heat up PBS to 50°C.

***Note:*** Pre-heating the PBS in a water-bath set to 50°C can help speed up this step if needed.

29. Add the PFA powder (10 g) to the PBS (250 mL) and heat to 60°C.

**CRITICAL:** PFA is harmful and carcinogenic. Handle with care. Wear gloves, and prepare the solution and aliquots in a fume hood.

30. Remove the solution from the hot plate, and add a few drops of NaOH until the PFA is fully dissolved and the solution is clear.
31. Manually pH to 7.2–7.4 using NaOH (or HCl).

**CRITICAL:** HCl is highly corrosive. Wear gloves, and only use in a fume hood.

32. Prepare 5 mL aliquots (sufficient to fix 10 slices) and store at −20°C for up to a year.

***Note:*** Avoid freeze-thaw. Once thawed, each aliquot should be used within the day, and discarded at the end of the day.

### Prepare stock solutions for immunohistochemistry

**Timing: 30 min, months to days before the recording day**

33. Prepare a 500 mL stock of 5% NaN_3_ PBS (dissolve 25 g of NaN_3_ in PBS). This solution can be diluted to prepare the 0.05% NaN_3_ PBS in which we store brain slices after fixation. Store this solution at 4°C until the day of use.

**CRITICAL:** NaN_3_ is highly toxic and competes for the O_2_ binding site on haemoglobin. Wear gloves and manipulate in a fume hood. Manipulate solutions containing NaN_3_ with gloves.

34. Prepare 100 mL of 1 ng/mL DAPI in 0.05% NaN_3_ PBS. Store this solution at 4°C until the day of use.

### Prepare stock solutions for OPC isolation with magnetic associated cell sorting (MACS)

**Timing: 1 h, up to a month before the experimental day**

35. Prepare a stock of 4 mg/mL DNase in ddH_2_O. Prepare 0.55 mL aliquots and store at −20°C until the day of use.

***Note:*** Avoid freeze-thaw. Once thawed, DNase aliquots can be kept at 4°C for up to one month.

36. Prepare ovomuccoid (see materials and equipment for the recipe).
  a. Combine DNase, BSA and trypsin inhibitor in DMEM.

***Note:*** Ovomuccoid can be kept at 4°C for up to one month.

### Prepare 1× bicarbonate-buffered aCSF for slicing and resting

**Timing: 40 min, up to one week before the experimental day**

37. Dilute the 10× stock in ddH_2_O and add glucose and MgCl_2_. See materials and equipment for the recipe.
38. Bubble the solution with 95% O_2_ / 5% CO_2_ for at least 20 min to set the pH to 7.2–7.4 and avoid CaCl_2_ precipitation.
39. Add CaCl_2_.
40. Add kynurenic acid to prevent excitotoxicity during slicing.

**CRITICAL:** Kynurenic acid does not readily dissolve. Prepare the slicing solution one day prior to use, and store at 4°C to allow the kynurenic acid time to dissolve completely. Alternatively, sonicate the solution for 5 min.

***Note:*** Slicing aCSF should be kept at 4°C, and discarded after one week. Discard the solution if it becomes cloudy, as this indicates CaCl_2_ precipitation.

### Prepare 1× bicarbonate- or HEPES-buffered aCSF for recording

**Timing: 40 min, up to one week before the experimental day**

41. Dilute the 10× stock in ddH_2_O and add glucose. See materials and equipment for the recipes.
  a. If required, add MgCl_2_.

***Optional:*** If using bicarbonate-buffered aCSF for recording, bubble the solution with 95% O_2_ / 5% CO_2_ for at least 20 min to set the pH to 7.2–7.4 and avoid CaCl_2_ precipitation.

42. Add CaCl_2_.
43. Manually adjust pH to 7.2–7.4 with NaOH.

***Optional:*** Omit step 43 if using bicarbonate-buffered aCSF for recording, as pH will already have been set by bubbling.

**CRITICAL:** MgCl_2_ should be omitted when focusing on NMDA receptor currents, as Mg^2+^ blocks NMDA receptors at holding potential (−74 mV).

**CRITICAL:** If recording NMDA receptor currents, add glycine (an NMDA receptor co-agonist) and strychnine (to block glycine receptors) to the recording aCSF. See steps 11 and 12, and materials and equipment for details.

***Note:*** Recording aCSF can be kept for up to one week at 4°C.

### Chloride a silver wire electrode and ground electrode

**Timing: 1 day, before the recording day**

44. Place a silver wire and the ground electrode pellet in bleach overnight (10–20 h).
45. Rinse the silver wire electrode and ground pellet in distilled water before placing them in the electrode holder and the recording bath, respectively.

**CRITICAL:** The recording electrode should be chlorided regularly, depending on the frequency of experiments. The electrode should be chlorided if it is not a uniform dull grey and shiny spots are showing, indicating that the chloride coating is wearing off. Similarly, the ground electrode should be checked regularly and chlorided when necessary.

### Prepare solutions for OPC isolation with MACS

**Timing: 1 h 30, on the experimental day**

46. Prepare 0.5% Bovine Serum Albumin (BSA) in PBS.
  a. Prepare ∼50 mL of BSA per brain.
  b. Filter the buffer with a vacuum filter, and keep the vacuum on until all bubbles have disappeared, as bubbles can block the columns.
  c. Store at 4°C until needed.

***Note:*** While BSA can sometimes be difficult to dissolve, 0.5% BSA should dissolve promptly, without vortexing.

***Note:*** While we typically prepare BSA on the experimental day, any remaining solution can be kept at 4°C for future use, for up to one month.

47. Prepare 30 mg/mL L-cysteine in DMEM. Keep at 4°C until needed. Discard any remaining L-cysteine after use.
  a. Prepare 0.2 mL L-cysteine per brain.
48. Prepare papain dissociation medium (see materials and equipment for the recipe).
  a. Prepare 1 or 2 mL per brain (see step-by step method details step 45).
  b. Combine papain and DNase in DMEM.
  c. Omit L-cysteine, which is only added 10 min prior to the experiment (see step-by step method details step 45a(i)).

***Note:*** Papain dissociation medium should be prepared the day of the experiment, and kept at 4°C until immediately prior to the experiment. Any remaining medium should be discarded.

## Key resources table

**Table:** 

REAGENT or RESOURCE	SOURCE	IDENTIFIER
**Antibodies**		

Anti-NG2 Chondroitin Sulfate Proteoglycan Antibody Dilution: 1:300	Millipore	Cat#AB5320; RRID:AB_91789
Anti-GFP antibody Dilution: 1:1000	Abcam	Cat#ab13970; RRID:AB_300798
Anti-Olig2 antibody Dilution: 1:300	Millipore	Cat#AB9610; RRID:AB_570666
CDP Antibody (M-222) (Cux1) Dilution: 1:100	Santa Cruz Biotechnology	Cat#sc-13024, RRID:AB_2261231
Goat anti-chicken IgY H&L (Alexa Fluor® 568) Dilution: 1:500-1:1000	Abcam	Cat#ab175477
Invitrogen Goat anti-Rabbit IgG (H+L) Highly Cross-Adsorbed Secondary Antibody, Alexa Fluor 647 Dilution 1:500-1:1000	Thermo Fisher	Cat#A-21245; RRID:AB_2535813

**Chemicals, peptides, and recombinant proteins**		

Kainic acid	Tocris	Cat#0222
NMDA	Tocris	Cat#0114
Strychnine hydrochloride	Sigma-Aldrich	Cat#S8753
Barium chloride dihydrate	Sigma-Aldrich	Cat#217565
Glycine	Sigma-Aldrich	Cat#G8898
NaCl	Sigma-Aldrich	Cat#S7653
KCl	Sigma-Aldrich	Cat# P3911
NaHCO_3_	Sigma-Aldrich	Cat#S5761
NaH_2_PO_4_	Sigma-Aldrich	Cat#S9638
CaCl_2_	VWR	Cat#21114
MgCl_2_	Fisher Scientific	Cat#15656060
D-Glucose	Sigma-Aldrich	Cat#G7528
Kynurenic acid	Sigma-Aldrich	Cat#K3375
HEPES	Sigma-Aldrich	Cat#H3375
BAPTA	Sigma-Aldrich	Cat#A4926
Potassium D-gluconate	Sigma-Aldrich	Cat#G4500
D-Gluconic acid	Sigma-Aldrich	Cat#G1951
CsOH	Sigma-Aldrich	Cat#516988
KOH	Sigma-Aldrich	Cat#P5958
NaOH	Sigma-Aldrich	Cat#06203
Mg_x_ATP	Sigma-Aldrich	Cat#A9187
Na_x_GTP	Sigma-Aldrich	Cat#G8877
K-Lucifer Yellow	Sigma-Aldrich	Cat#L0144
Ultra-pure low-melting point agarose	Fisher Scientific	Cat#16520050
Bleach	n/a	n/a
DAPI	Sigma-Aldrich	Cat#D9542
NaN_3_	Fisher Scientific	Cat#10592211
PBS	n/a	n/a
Triton-X 100	Sigma-Aldrich	Cat#T8787
Goat serum	Sigma-Aldrich	Cat#G9023
Fluoromount-G	SouthernBiotech	Cat#0100-01
Paraformaldehyde	Fisher Scientific	Cat#P/0840/53
Hydrochloric acid	Sigma-Aldrich	Cat#320331
Bovine serum albumin	Sigma-Aldrich	Cat#A4919
Papain from papaya latex, buffered aqueous suspension, 2× crystallized, 16–40 units/mg protein	Sigma-Aldrich	Cat#P3125
Deoxyribonuclease (DNase) I from bovine pancreas, type IV	Sigma-Aldrich	Cat#D5025
L-Cysteine	Sigma-Aldrich	Cat#C7352
DMEM	Invitrogen	Cat#41966029
Trypsin inhibitor from *Glycine max* (soybean)	Sigma-Aldrich	Cat#T9003
Β-Mercaptoethanol	Sigma-Aldrich	Cat#M6250
Ethanol	Sigma-Aldrich	Cat#32221-M

**Critical commercial assays**		

Myelin Removal Beads II, human, mouse, rat	Miltenyi Biotec	Cat#130-096-733
CD140a (PDGFRα) MicroBead Kit, mouse	Miltenyi Biotec	Cat#130-101-502
RNAeasy Micro Kit	QIAGEN	Cat#74004
SMARTer Stranded Total RNA-seq Kit v2 – Pico Input Mammalian	Takara Clontech	Cat#634411

**Experimental models: organisms/strains**		

Mouse: NG2-EYFP	Prof Jacqueline Trotter; Karram et al., 2008	n/a
Mouse: PdgfrαCre^ERT2^	Prof. William D. Richardson ; Rivers et al., 2008	MGI:3832569
Mouse: Ai9(RCL-tdT)	Madisen et al., 2010	JAX:007909
Mouse: C57BL/6 wild-type	Charles River Laboratories	C57BL/6NCrl, RRID:IMSR_CRL:27

**Software and algorithms**		

pCLAMP10.3	Molecular Devices	n/a
MATLAB	MathWorks	URL: https://uk.mathworks.com/
Cell capacitance analysis	Written in house	n/a
Na_V_ current analysis	Written in house	n/a
GraphPad Prism	GraphPad Software	URL: https://www.graphpad.com/scientific-software/prism/
ImageJ	NIH	URL: https://fiji.sc/ or https://imagej.nih.gov/ij/
LAS AF/LAS X	Leica	URL: https://www.leica-microsystems.com/products/microscope-software/details/product/leica-las-x-ls/
Trim Galore!	Babraham Bioinformatics	URL: https://www.bioinformatics.babraham.ac.uk/projects/trim_galore/
TopHat 2.1.1	Center for Computational Biology at Johns Hopkins University	URL: https://ccb.jhu.edu/software/tophat/index.shtml
R Bioconductor DESeq2	Love et al., 2014	URL: https://bioconductor.org/packages/release/bioc/html/DESeq2.html
DAVID	Laboratory of Human Retrovirology and Immunoinformatics	URL: https://david.ncifcrf.gov/
REVIGO	Supek et al., 2011	URL: http://revigo.irb.hr

**Other**		

Dissection scissors (large and small)	n/a	n/a
Glass beakers	n/a	n/a
Glass petri dish	n/a	n/a
Forceps (curved and straight)	n/a	n/a
Spatula (large and small)	n/a	n/a
Platinum-coated commercial razor blades	Supermax Blue Diamond	n/a
Cyanoacrylate glue (superglue)	Loctite	n/a
Polystyrene ice box	n/a	n/a
Hot plate	Stuart	Cat#UC152
Falcon® 100 mm TC-treated Cell Culture Dish	Corning	Cat#353003
4-well plate	Thermo Scientific	Cat#176740
95% O_2_ / 5% CO_2_ cylinder	n/a	n/a
100% O_2_ cylinder	n/a	n/a
Vibrating microtome	Leica	Cat#VT1200S
Resting chamber	Lab made	n/a
Glass Pasteur pipette (with the thin end cut off)	Poulten & Graf	Cat#D812
Vertical two-step puller	Narishige	Cat#PC-100
Microforge	Narishige	Cat#MF-830
Thick-walled borosilicate glass capillaries with filament, type GC150F-10	Harvard Apparatus	Cat#30-0057
Bunsen burner	n/a	n/a
Harp	Lab made	n/a
Disposable scalpel	Swann-Morton	Cat#05XX
Ag/AgCl ground pellet	Warner Instruments	Cat# 64-1314
Silver wire	Sigma-Aldrich	Cat# 327034
Electrode holder	Molecular Devices	Cat#HL-1-17
Headstage	Axon Instruments	Cat#CV-201
Amplifier	Axon Instruments	Cat#Axopatch200
Digitizer	Axon Instruments	Cat#Digidata 1440A
Anti-vibration air table	n/a	n/a
Faraday cage	Lab made	n/a
Upright fixed-stage infrared (IR) differential interference contrast (DIC) microscope, with a 4/5× objective and a 40×/63× water immersion objective	Olympus	Cat#BX51WI
Recording chamber	Lab made	n/a
Halogen lamp	EXFO	Cat#X-Cite 120
Excitation/emission filters	n/a	n/a
Micromanipulators	Luigs & Neumann	n/a
Gravity-fed perfusion system	Lab made	n/a
Microscope-attached IR-CCD camera	Watec	n/a
TV monitor	n/a	n/a
Computer	n/a	n/a
Rotating shaker	Stuart	Cat#SSM1
Polysine adhesion slides	Thermo Scientific	Cat# 10219280
24-well plates	Corning	Cat#353047
MACS Multistand	Miltenyi	Cat#130-042-303
MiniMACS Separator	Miltenyi	Cat#130-042-102
MidiMACS Separator	Miltenyi	Cat#130-042-302
Benchtop centrifuge	n/a	n/a
7 mL Bijou Container	n/a	n/a
15 mL Tubes	n/a	n/a
50 mL Tubes	n/a	n/a
70 μm Cell strainers	STARLAB	Cat#CC8111-0072
Disposable vacuum filter	Corning	Cat#10016110

## Materials and equipment

### Electrophysiological setup

The hardware and software for whole-cell patch clamp recordings listed in the key resources table above are examples based on the set up we have in the lab. However, there are several other suitable manufacturer and models. Here, we list the basic components of the electrophysiological set up and hardware.

• An upright infrared differential interference contrast (IR-DIC) microscope with a light source, appropriate filters for fluorescent imaging (if needed), a 4/5× objective, and a 40/63× objective (available from, for instance, Olympus, Leica, and Zeiss).
• An anti-vibration air table.
• A Faraday cage.
• A headstage, amplifier, and digitizer (available from Axon Instruments, HEKA, Sutter, and Cambridge Electronic Design).
• Micromanipulators (available from, for instance, Luigs & Neumann, Scientifica, Sutter, and Thorlabs).
• A perfusion system (gravity-fed or with a peristaltic pump, for instance).
• A recording chamber for brain slices and a 'harp' (a thin U-shaped non-conductive metal strung with nylon, for instance) to hold the brain slice steady during recordings.
• An IR-CCD camera.
• A computer, computer monitor and TV screen.
• A source of 100% O_2_ (or 95% O_2_ / 5% CO_2_ if using bicarbonate-buffered recording aCSF).

**Table:** 10× bicarbonate-buffered aCSF for slicing and resting (see key resources table for more details on reagents)

Reagent	Final concentration (10× stock)	Amount	Final concentration (1× working solution)
NaCl	1.24M	72.47 g	124 mM
NaHCO_3_	260 mM	21.84 g	26 mM
NaH_2_P0_4_ [1M solution]	10 mM	10 mL	1 mM
KCl [1M solution]	25 mM	25 mL	2.5 mM
ddH_2_O	n/a	Complete to 1L	n/a

**CRITICAL:** 10× bicarbonate-buffered aCSF can be used for up to a month. The solution should be stored at 4°C and should be discarded if any precipitate forms.

**Table:** 1× bicarbonate-buffered aCSF for slicing and resting

Reagent	Final concentration (1× working solution)	Amount
10× solution	1×	100 mL
D-glucose	10 mM	1.8 g
MgCl_2_ [1M solution]	2 mM	2 mL
CaCl_2_ [1M solution]	2.5 mM	2.5 mL
ddH_2_O	n/a	Complete to 1L

**For the slicing and resting solution only:**		

Kynurenic acid	1 mM	0.189 g
**Total**		**1L**

***Note:*** The expected osmolarity of this solution is 315–330 mOsm.

**CRITICAL:** 1× slicing and resting solution can be made up weekly and stored at 4°C. pH should be equilibrated at the start of each experimental day with at least 30 min of bubbling with 95% O_2_ / 5% CO_2_.

***Optional:*** Bicarbonate-based aCSF can also be used for recordings. For that purpose, prepare a separate 1× stock and omit kynurenic acid. Make sure to add the correct MgCl_2_ concentration for the experimental design.

**Table:** 10× HEPES-buffered aCSF for recording

Reagent	Final concentration (10× stock)	Amount	Final concentration (1× working solution)
NaCl	1.44M	84.15 g	144 mM
HEPES	100 mM	23.85 g	10 mM
NaH_2_P0_4_ [1M solution]	10 mM	10 mL	1 mM
KCl [1M solution]	25 mM	25 mL	2.5 mM
ddH_2_O	n/a	Complete to 1L	n/a

**CRITICAL:** 10× HEPES-buffered aCSF can be used for up to a month and should be stored at 4°C. Discard the stock solution if any precipitate is observed.

**Table:** 1× HEPES-buffered aCSF for recording

Reagent	Final concentration (1× working solution)	Amount
10× solution	1×	100 mL
D-glucose	10 mM	1.8 g
MgCl_2_ [1M solution]	0–2 mM	0–2 mL
CaCl_2_ [1M solution]	2.5 mM	2.5 mL
ddH_2_O	n/a	Complete to 1L
**Total**		**1L**

***Note:*** The osmolarity of HEPES-buffered recording aCSF is expected to be 315–330 mOsm.

**CRITICAL:** 1× recording solution can be stored at 4°C and used for up to a week.

***Optional:*** If recording NMDA receptor currents, add the following to the recording aCSF immediately prior to recording:

**Table:** 

Reagent	Final concentration (1× working solution)	Amount
Glycine [1M solution]	0.1 mM	0.1 mL
Strychnine hydrochloride [50 mM solution]	0.005 mM	0.1 mL
1× HEPES-buffered recording aCSF (or alternatively bicarbonate-buffered aCSF)	n/a	1L
**Total**		**1L**

**CRITICAL:** Strychnine hydrochloride is acutely toxic, and ingestion or inhalation can be fatal. Gloves should be worn when manipulating any solutions containing strychnine.

***Optional:*** If recording in adult grey matter, add the following to the recording aCSF immediately prior to recording:

**Table:** 

Reagent	Final concentration (1× working solution)	Amount
Barium chloride [1M solution]	0.2 mM	0.2 mL
1× HEPES-buffered recording aCSF (or alternatively bicarbonate aCSF)	n/a	1L
**Total**		**1L**

**CRITICAL:** Barium chloride dihydrate is toxic. When making up stock solution from powder, wear gloves, a mask, and only handle in a fume hood. Gloves should be worn when manipulating solutions containing barium.

**Table:** Internal solution

Reagent	Final concentration	Amount
Cs-gluconate or K-gluconate [1M solution]	130 mM	3.25 mL
NaCl [4M solution]	4 mM	0.025 mL
CaCl_2_ [1M solution]	0.5 mM	0.0125 mL
HEPES [1M solution]	10 mM	0.250 mL
BAPTA	10 mM	119.1 mg
Mg_x_ATP	4 mM	50.72 mg (from free acid molecular weight)
Na_x_GTP	0.5 mM	6.54 mg (from free acid molecular weight)
Lucifer Yellow	~2 mM	25 mg
ddH_2_O	n/a	Complete to 25 mL
**Total**		**25 mL**

***Note:*** We add 25 mg Lucifer Yellow (one vial) into 25 mL of internal solution, as this is the simplest method, and an exact final concentration is not critical.

***Note:*** The expected osmolarity for this internal solution is 290–300 mOsm.

**CRITICAL:** Internal solution should be aliquoted and stored at −20°C for up to one year. The aliquot size should correspond to the amount of internal solution needed for one day of recording (typically between 0.1 mL and 0.5 mL). Once thawed, internal solution should be kept on ice and protected from light, and should be discarded at the end of the day.

***Alternatives:*** Neurobiotin, Alexa Fluor dyes or fluorescent-conjugated dextran can be used as alternatives to Lucifer Yellow.

***Alternatives:*** Cs- or K-methanesulfonate can be used instead of Cs- or K-gluconate in this recipe. Cs- or K-chloride can also be used, but a high intracellular chloride concentration will affect the chloride reversal potential, and may result in inhibitory inputs depolarizing the cells.

**Table:** Papain dissociation medium

Reagent	Final concentration	Amount
Papain	4%	0.160 mL
DNase [4 mg/mL]	0.04 mg/mL	0.040 mL
DMEM	n/a	3.4 mL

**Add 10 min before use**		

L-cysteine [30 mg/mL]	3 mg/mL	0.4 mL
**Total**		**4 mL**

**Table:** Ovomuccoid

Reagent	Final concentration	Amount
DNase [4 mg/mL]	0.04 mg/mL	0.5mL
BSA	0.5 mg/mL	25 mg
Trypsin inhibitor	1 mg/mL	50 mg
DMEM	n/a	Complete to 50 mL
**Total**		**50 mL**

## Step-by-step method details

### Preparing acute brain slices

**Timing: 2 h**

Here, we describe how to prepare acute brain slices from embryonic (E13-E20) or postnatal brains (≥P0) for patch clamp recording. We use both male and female animals.

1. Before you begin, prepare for the dissection and slicing.
  a. Bubble the slicing aCSF with 95% O_2_ / 5% CO_2_ for at least 30 min (to set pH to 7.2–7.4), on ice.
  b. Calibrate the vibratome blade according to the manufacturer’s instructions.
  c. Place the slicing chamber in the freezer for at least 5 min.
    i. Once it is cooled, place the slicing chamber on the vibratome, and fill the outer chamber with ice.
  d. Once you have bubbled the slicing aCSF for 30 min, fill the chamber with ice-cold slicing aCSF, and continuously bubble the slicing solution with 95% O_2_ / 5% CO_2_ until the last slice is cut (step 6), so that the pH of the aCSF is maintained throughout the procedure.
  e. Cool two beakers (one 10 mL and one 50 mL) and a glass petri dish on ice.
  f. Fill the resting chamber with slicing aCSF and continuously bubble with 95% O_2_ / 5% CO_2_ until the end of the recording day.
  g. Fill the two beakers with bubbled slicing aCSF (after the 30 min of bubbling).

***Optional:*** When slicing from young postnatal brains (up to P20), we suggest using a block of agarose to support the brain while slicing. This is also useful when slicing from brains regions where the required slicing angle is not readily maintained (such as the cerebellum), but is usually not needed when cutting coronal forebrain slices from adult mice, as the brain is stiff enough to remain stable when slicing at low speed. If you choose to use agarose, cut out a cube of 5% agarose and glue it on the vibratome slicing platform. Make sure that the brain will rest against a flat surface (Figure 1).

***Optional:*** If preparing embryonic slices, dissolve 1 g low melting point agarose in 50 mL of 1× recording aCSF by bringing the agarose to boiling point using a hot plate. Then, cool down the 2% agarose to avoid damaging the tissue when embedding, but do not allow it to set. This can be done while the slicing aCSF is bubbling (step 1a).

2. Humanely sacrifice an animal in accordance with local regulations.

***Note:*** When preparing embryonic slices, sacrifice the pregnant female first, then dissect the embryos.

3. Remove the head and immerse it in the 50 mL glass beaker containing bubbled slicing aCSF.
4. Dissect the brain.
  a. Cut the skin along the midline, exposing the skull, and use it to hold the head by folding it under the head.
  b. Make a cut between the eyes, make lateral cuts in the skull anterior to posterior, and remove the skull. Alternatively, make a cut near the midline above the hindbrain, peel off the skull above the brainstem and cerebellum, and then make lateral cuts in the skull above the forebrain, posterior to anterior, and peel off the skull.
  c. Carefully extract the brain using a small spatula. For best results, dip the spatula in slicing aCSF first.
  d. Immerse the brain in the 10 mL glass beaker containing bubbled slicing aCSF.
  e. Fill the glass petri dish with bubbled aCSF and transfer the brain into the petri dish using the large spatula. Keep the petri dish on ice.

***Optional:*** For postnatal brains, dissect the meninges, and then dissect the region of interest (for instance, the forebrain).

***Optional:*** For embryonic slices, fill one well in a 4-well plate halfway with the melted agarose. Place a dissected brain in the well and add more agarose to fill the well. Ensure that the brain is correctly positioned for the required slice orientation (coronal/sagittal/transverse). Repeat this procedure for each brain. Place the plate on ice or in the fridge to allow the agarose to set rapidly. We recommend preparing embryonic slices with two people: one person should dissect while the second person should embed the brains.

5. Glue the brain to the vibratome slicing platform in the correct orientation for the required slices (Figure 1). Consult a brain atlas to determine the right slicing orientation to keep axonal fibers intact in the region of interest.

***Optional:*** For embryonic slices, once the agarose has set, cut a block around the embedded brain and glue this block to the vibratome slicing platform (Figure 1).

6. Cut 225 μm–325 μm thick slices. Cut enough slices for a full day of recording.

***Note:*** Slice thickness is a compromise between axonal integrity (thicker slices allow for better axonal preservation) and cell visibility, especially when patching small cells and using fluorescent reporter lines. For instance, when recording from OPCs in postnatal brains, we typically use 225 μm thick slices. For embryonic slices, we recommend cutting 325 μm thick slices, as embryonic slices are fragile, and slice integrity is not always maintained in 225 μm thick slices. Embryonic slices are more transparent, as they are not myelinated, and thus, cell visibility is better, even with thicker slices.

7. Transfer each slice into the resting chamber using the glass Pasteur pipette.
8. Allow the slices to rest for 1 h before recording.

**CRITICAL:** Time is crucial when preparing acute brain slices. If the delay between sacrificing an animal and placing the brain in the ice-cold aCSF-filled vibratome chamber is too long, the slices may not be healthy enough, which will make patch clamp recordings particularly difficult. Troubleshooting 1.

### Whole-cell patch clamp recording in reporter mice with dye loading

**Timing: 8–10 h**

Here, we describe how to record from OPCs in acute brain slices from fluorescent reporter mice such as NG2-EYFP, NG2-dsRed, or PdgfrαCre^ERT2^:tdTomato using whole-cell patch clamp. OPCs can be identified in acute brain slices without any fluorescent label, but this requires a trained eye, and post-hoc immunohistochemistry to confirm cell identity (for more detail on identifying oligodendrocyte lineage cells for patch clamp, see Agathou and Káradóttir (2019)). The following steps can be used for recording non-neuronal cells in acute brain slices with or without the use of reporter mice.

**Figure 2:**
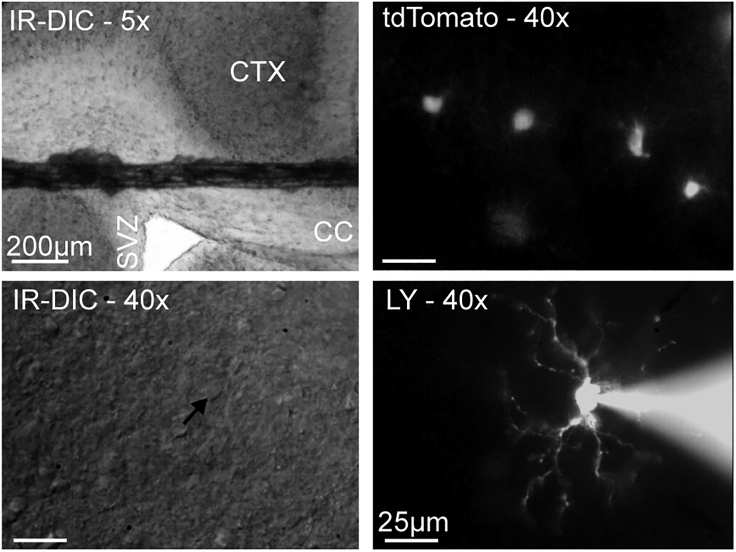
Choosing an OPC for whole-cell patch clamp The top left panel shows a coronal forebrain slice imaged with a 5× IR-DIC objective. The cortex (CTX), corpus callosum (CC) and subventricular zone (SVZ) are labeled on the slice. A harp string is holding the slice in place. Using a low magnification objective to visualize the slice first is useful to choose the region of interest. The top right panel shows PdgfrαCre^ERT2^:tdTomato cells in the cortex imaged with a 40× water immersion objective. Identifying potential tdTomato+ cells to patch is the second step to pick a cell to record. The bottom left panel shows a cortical OPC (black arrow head) under IR-DIC. This is a good cell for patch clamp, as it has smooth but clear outlines, is not too deep in the slice, and has good access. The bottom right panel shows a patched OPC filled with Lucifer Yellow (LY). Scale bar for the 5× image: 200 μm. Scale bar for the 40× images: 25 μm.

**Figure 3:**
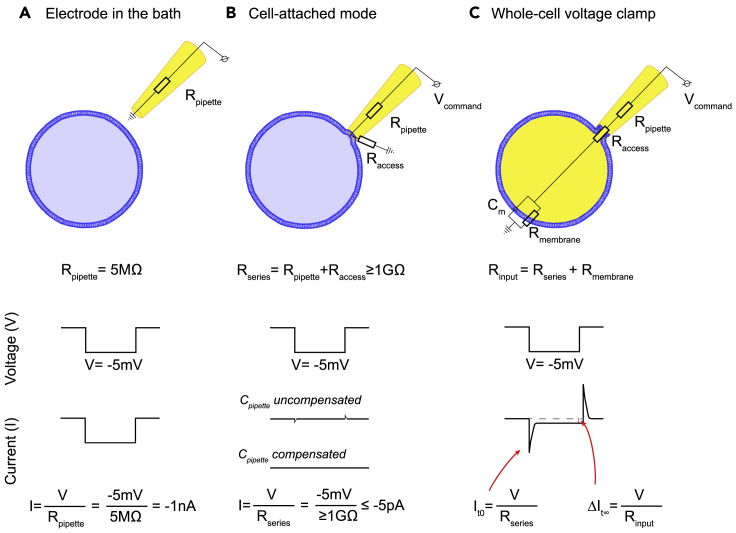
Sealing and opening a cell (A) When the electrode is in the bath, applying a −5 mV voltage pulse leads to current flow across the pipette resistance (R_pipette_) only. This current can be calculated using Ohm’s law, *V=IR*. (B) Once a giga-ohm seal is achieved, the same voltage step will elicit current flow across the pipette resistance and the seal resistance (R_access_). We use Ohm’s law to calculate this current, but it is near 0, as R_access_ is very large. In most cells, the formation of a giga-ohm seal is not instantaneous. It can be monitored by tracking the change in current (and resistance) with (A) as a starting point and (B) as the end point. (C) Once the cell is open, current flows across the pipette resistance, access resistance (adding up to the series resistance, R_series_), the membrane resistance (R_membrane_) and the membrane capacitance (C_m_). Analysis of the capacitive current elicited by a −5 mV step allows the calculation of R_series_, R_membrane_ and C_m_ (see Figure 8).
16. Adjust the pipette offset current to zero on the amplifier.
17. Apply positive pressure to prevent debris from collecting at the tip of the electrode. We use a lab-made system where we apply positive pressure by blowing into a 1 mL syringe connected to the headstage by tubing.

***Note:*** This initial positive pressure can be achieved by blowing into the syringe and locking the valve connecting the syringe to the tubing.

9. Prepare the pipettes and patch clamp set up for the day.
  a. Fire-polish both ends of glass capillaries using a Bunsen burner (unless you are buying in fire-polished glass capillaries).
  b. Use the fire-polished capillaries to pull glass electrodes using an electrode puller. For OPCs, we recommend pulling electrodes with a 5–7 MΩ resistance.
    ***Note:*** It is possible to use electrodes with resistances as low at 4.5 MΩ to patch OPCs, but this is very challenging, as they tend to have almost the same diameter as most OPCs. A resistance of 5–7 MΩ is a good compromise between ease of seal formation, ease of cell opening, and series resistance during the recording. Troubleshooting 2 and 4.
  c. Thaw an aliquot of internal solution and keep it on ice and protected from light.
    ***Note:*** To record from OPCs, we typically use Cs-gluconate or K-gluconate based internal solutions. Cs-gluconate allows for more stable recordings than K-gluconate, as it blocks some K^+^ channels (Hille, 2001). We tend to use Cs-gluconate when recording NMDA currents as these currents can be very small, and could be masked by K^+^ channel noise. Cs-gluconate internal is also recommended to record voltage-gated Na^+^ currents. When recording K^+^ currents, a K-gluconate based internal solution is more appropriate.
  d. Thaw an aliquot of 4% PFA and keep it at room temperature (19°C–23°C) for fixing slices after recording.
    **CRITICAL:** PFA is harmful. Handle with care. Wear gloves and use in a fume hood or under an air extraction vent.
  e. Turn on all the electrical equipment.
  f. Oxygenate the recording aCSF with 100% O_2_ (when using a HEPES-buffered recording solution; for a bicarbonate-buffered aCSF, bubble with 95% O_2_ / 5% CO_2_) and start running the solutions through the gravity-fed perfusion. Ensure that the lines are free of air bubbles. Troubleshooting 6. Bubble the solutions throughout the recordings.

10. Transfer a slice from the resting chamber into the recording chamber using a glass Pasteur pipette.
  a. Use a harp (a thin U-shaped non-conductive metal strung with nylon, for instance) to hold the slice in position at the bottom of the recording chamber.
11. Use the 4/5× objective to locate the slice under the microscope, and identify the region of interest (Figure 2).

12. Switch to a 40×/63× immersion objective with IR-DIC optics (Figure 2).
  a. Use the fluorescence to identify reporter-expressing OPCs.
  b. Switch back to brightfield to identify a good OPC to attempt patch clamp.
    ***Note:*** Under IR-DIC, good OPCs appear smooth, with a soft and clear outline, while shiny cells with a hard outline should be discarded as they are difficult to seal. Round cells with a visible nucleus should be avoided, as they are dying cells. Cell location (proximity to other cells, depth in the slice) cannot be assessed when using fluorescence only. Thus, it is best to alternate between fluorescence and brightfield until a suitable cell is identified (healthy, with good access and good slice depth).
    ***Note:*** When using fluorescent reporters under the NG2 promoter, we avoid cells adjacent to blood vessels, as pericytes also express NG2. Pericytes are covered by vascular basement membrane and are therefore much more difficult to seal than OPCs, requiring smaller pipette tips and/or pre-incubation of slices in collagenase, as well as a longer time to seal (Mishra et al., 2014; Peppiatt et al., 2006). Nonetheless, the current-voltage relationship (IV) differs between OPCs and pericytes, with pericytes displaying a different capacitive current and potassium conductance (Sakagami et al., 1999). It is therefore particularly important to apply a voltage step protocol (see step 27) at the beginning of each recording, as each cell type have their specific ion channel expression and IV. We recommend consulting several IVs for the cell type of interest before starting patch clamp experiments.
  c. Mark the location of the cell on the screen to easily locate it once the electrode is in the bath.

13. Fill a glass pipette with internal solution and fit it in the electrode holder on the micromanipulator headstage so that the chlorided electrode is in contact with the internal solution.
14. Raise the objective so that it is still in contact with the bath solution, but there is enough space to lower the electrode into the bath.
15. Lower the electrode into the bath, and begin applying a ±5 mV (or 10 mV) 15 ms voltage pulse every 30 ms. Display the resulting current on your computer monitor using Clampex (or another appropriate software). Continue these pulses until you have achieved whole-cell configuration (Figure 3).

18. Find the electrode under the microscope, and lower the electrode and objective until you are just above the cell of interest.
19. Increase the positive pressure by continuously blowing into the syringe as you approach the cell.
20. When the electrode contacts the cell, a dimple should appear and resistance should slightly increase while current decreases. Switch from positive pressure to negative pressure (achieved by suction into the syringe).
21. Set the holding potential to −60 mV on the amplifier.

***Note:*** We measured a junction potential of −14 mV between our internal solutions and recording aCSF. Corrected for junction potential, a holding potential of −60 mV on the amplifier is a −74 mV holding potential. However, junction potential should be measured experimentally for each internal-external solution combination, and the holding potential on the amplifier may need to be adjusted.

22. Continue to apply negative pressure until a giga-ohm seal is achieved. Monitor the formation of a seal on Clampex, using the continuous voltage pulses and paying close attention to the resistance, which should reach at least 1 GΩ, and the resulting reduction in current (Figure 3B). Troubleshooting 2 and 3.

***Note:*** Different OPCs seal at different rates. For some cells, simply switching from positive pressure to light negative pressure is enough to obtain a giga-ohm seal. However, other cells require sustained negative pressure for 30 s–1 min before a seal is achieved. The negative pressure will have to be adapted for each cell. This can be gaged by monitoring the increase in resistance and decrease in current.

23. Once a giga-ohm seal is achieved, compensate pipette capacitance on the amplifier (Figure 3B). Follow the instructions in the manufacturer’s manual to apply compensation.
24. Apply short, hard, negative pressure pulses to open the cell. Once the cell has opened, a membrane capacitive current will be visible in response to the voltage pulses (Figure 3C). In addition, the Lucifer Yellow-containing internal solution will be visible in the cell (Figure 2). Troubleshooting 4 and 5.

***Note:*** As with sealing, different OPCs will require different negative pressure to open. Be cautious, as too much negative pressure will break the seal and the cell will have to be discarded. However, some cells will require many pulses before opening. OPCs in slices from aged mice are typically harder to open than OPCs in slices from neonates. Troubleshooting 4 and 5.

25. Apply a 50 ms ± 5 mV voltage pulse. From this pulse, we calculate R_s_, membrane capacitance (C_m_) and membrane resistance (R_m_). See quantification and statistical analysis for more detail.
26. Compensate series resistance (R_s_) (according to the instructions in the manufacturer’s manual). If recording large current changes, it is critical to compensate for R_s_. Monitor R_s_ throughout the recording by regularly applying a 50 ms ±5 mV voltage pulse.

***Note:*** We recommend recording from cells with an R_s_ ≤ 25 MΩ. However, if R_s_ is larger than 25 MΩ, but the cell has a stable baseline and there is minimal leak current (see step 28), recording from OPCs with R_s_ ≤ 40 MΩ is still acceptable. If R_s_ is larger than 40 MΩ, the cell should be discarded. Similarly, if R_s_ changes by more than 20% throughout the recording, the cell should be discarded.

27. Apply a protocol delivering voltage pulses to step the membrane potential from −120 mV to +40 mV (using 10 mV or 20 mV steps) and record the current responses. From this protocol, you can determine the ion channel expression in the recorded cell. While we found that OPCs are heterogeneous with respect to their voltage-gated ion channel expression (Spitzer et al., 2019), this profile can be used to distinguish them from more mature oligodendrocytes if necessary (see Agathou and Káradóttir, 2019). See quantification and statistical analysis for more detail on quantifying voltage-gated Na^+^ channel current responses.

***Optional:*** When recording in the grey matter of adult animals, we recommend switching to a recording aCSF containing BaCl_2_ after the first voltage step protocol. This will block potassium conductance, which has been shown to increase with age (Maldonado et al., 2013). This is particularly important if comparing bath applied agonist responses in neonates and adults; however, avoid using BaCl_2_ when investigating K^+^ currents.

28. Apply any stimulation or voltage protocols and drugs required to answer your research question. For instance, in Spitzer et al., 2019, we typically applied NMDA, then kainate, as we were studying changes in NMDA and AMPA/kainate receptors with age and brain region. See quantification and statistical analysis for more detail on quantifying receptor agonist responses.
  a. Once you have recorded a stable baseline, bath apply NMDA first. If the cell responds, keep NMDA on until a stable peak is reached or the response begins to recover (indicating receptor desensitization). Wash out with recording aCSF. If the cell does not respond, leave NMDA on for 4–5 min to confirm that the lack of response is due to an absence of NMDA receptors, and not receptor desensitization due to a decrease in perfusion flow rate. Troubleshooting 6.
  b. Once the cell has fully recovered from an NMDA response and you have acquired a stable baseline, bath apply kainate. Keep kainate on until a stable peak is reached.

***Note:*** If the recording becomes unstable and does not recover within a few minutes, the seal may have broken, or the cell may be dying. The recording should be stopped, as the results may become uninterpretable. Similarly, if the leak current is larger than −400 pA (in Cs-gluconate; −200 pA in K-gluconate), the recording should be stopped.

**Figure 4:**
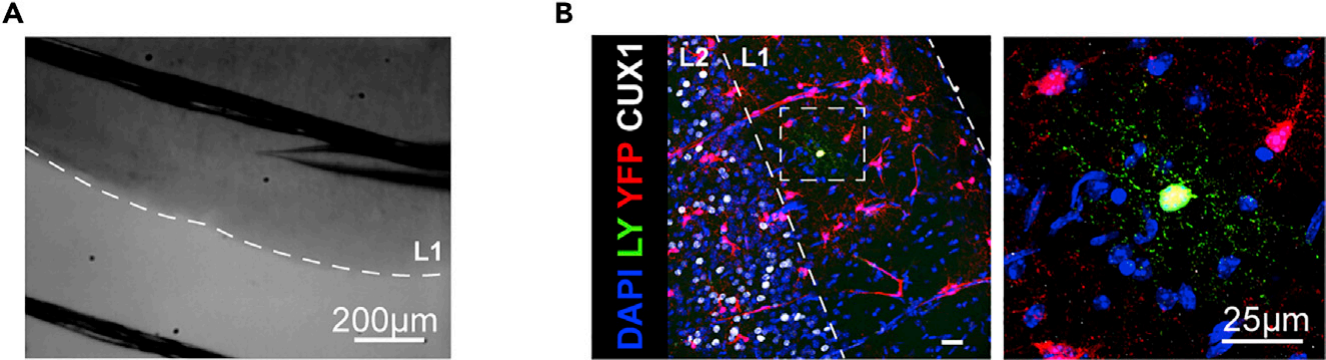
Post-hoc immunohistochemistry for cell identification and cell location (A) Image taken with a 5× IR-DIC objective after patch clamp, to record cell location for post-hoc immunohistochemistry. Scale bar 200 μm. (B) Following patch clamp of NG2-EYFP cells, and dye loading with Lucifer Yellow (LY), we fixed the slices and performed post-hoc immunohistochemistry against EYFP to confirm cell identify, and Cux1, a marker of cortical layers 2/3 to locate the cells. This image shows a cell in cortical layer 1. Scale bar 25 μm. (B) is reprinted from Spitzer et al., 2019 (with permission according to a Creative Commons Attribution License (CC BY)).
30. Remove the electrode from the cell, trying to keep the cell in the slice. Depolarizing the cell to 0 mV may help.

***Note:*** For cells with a high seal resistance (>10 GΩ), we recommend slowly pulling the pipette away and upwards. A thread of membrane should appear between the electrode and soma, and detach from the cell body, leaving it intact in the slice. If this is not sufficient, or for cells with lower seal resistance, positive pressure or moving the pipette sideways may help to detach the electrode from the cell body. When neither of these methods work, pulling the pipette away quickly, or gently taping the stage may help, although the dye may leak out. In addition, long recordings make it significantly more difficult to keep the cell in the slice when removing the electrode.

29. Once the recording is done, capture an image of the Lucifer Yellow filled cell (Figure 2). Switch back to the 4/5× objective to capture an image of the pipette location on the slice (Figure 4A). Record this in your lab book. This will help to locate the cell when imaging post-hoc antibody staining.

31. If attempting your next cell on the same slice, repeat steps 12–30. We recommend changing slices approximately every hour, as slice health will deteriorate in HEPES-buffered recording aCSF. If using drugs that are difficult to wash out, or bind irreversibly, change slices after each cell.
32. If changing slices, repeat steps 10–30.
33. Note the slice orientation in your lab book. It may be necessary to make a mark on symmetrical slices. Use a disposable scalpel to do so. This will be helpful for post-hoc immunohistochemistry.
34. Immediately fix the finished slice in 4% PFA in a 24-well plate for one hour at room temperature (19°C–23°C) on a rotating shaker, or overnight (10–20 h) at 4°C, on a rotating shaker. After fixing the slices, wash 3 × 30 min in PBS and store at 4°C in 0.05% NaN_3_ in PBS.

**Pause point:** When kept in these conditions, fixed slices can be used for up to a month after patch clamp with successful recovery of Lucifer Yellow filled cells. However, for best results, we recommend performing immunohistochemistry within a week following patch clamp and slice fixation.

### Post-hoc immunohistochemistry for cell identification and cell location

**Timing: 2–3 days**

Here, we briefly describe how to use immunohistochemistry to confirm the identity of patched cells, or obtain more information on the cells, such as location (with layer specific markers for instance) or proliferative state. As this has previously been described in great detail (please consult Karadottir and Attwell (2006) for an in-depth protocol), we simply summarize the key steps.

35. Wash the slices for 15 min in PBS.
36. Incubate the slices in blocking solution (10% goat or donkey serum and 0.5% Triton-X 100, in PBS) at room temperature (19°C–23°C) on a rotating shaker for 4 h.
37. Incubate the slices with primary antibodies in PBS overnight (10–20 h), at room temperature (19°C–23°C) on a rotating shaker.

***Note:*** To confirm OPC identity, we recommend staining with an antibody against your reporter (this is not always necessary, as the endogenous signal may still be detected post-fix; however, EYFP fluorescence is quenched by PFA fixation, so using an antibody is important to detect the cells in EYFP reporter mice), or anti-NG2 or anti-Pdgfrα, two OPC markers (Figure 4B). In addition, staining with anti-Olig2 (an oligodendrocyte lineage marker) can help confirm that the cell is an OPC rather than a pericyte (as pericytes are also NG2+). Appropriate markers should be used if performing this protocol with a different cell type.

***Note:*** To confirm cell location, a range of markers can be used, depending on the region of interest. For instance, in Spitzer et al., 2019, we located cells within specific cortical sections. To do so, we used Cux1, which labels cells in cortical layers 2/3, and CTIP2, which labels cells in cortical layers 5/6 (Figure 4B).

38. Wash 3 × 30 min in PBS.
39. Incubate the slices with secondary antibodies in PBS at room temperature (19°C–23°C) on a rotating shaker for 5 h.

**Pause point:** Alternatively, the secondary antibody incubation can be done overnight (10–20 h) at 4°C on a rotating shaker.

40. Wash 2 × 30 min in PBS.
41. Incubate with DAPI for 20 min.
42. Wash 30 min in PBS.
43. Mount the slices on glass microscope slides.
44. Image the patched cells using a confocal microscope (Figure 4B).

***Note:*** To locate Lucifer Yellow-filled cells in brain slices, we recommend using a low magnification objective (20×) and scanning the slice through the eyepiece. Recording the cell location after patch clamp should help to confine the search region.

### Isolation of OPCs for bulk RNA sequencing

**Timing: 5 h**

Here, we describe how to isolate OPCs from wild-type C57BL/6 mice at different timepoints using Magnetic Associated Cell Sorting (MACS). We briefly list our pipeline for RNA sequencing following cell isolation, but do not detail it, as this is not the focus of this protocol (and thus, is not included in the timing for this step).

**Pause point:** This section of the protocol is independent from the preparation of acute brain slices, whole-cell patch clamp and post-hoc immunohistochemistry sections. Thus, it can be performed at any given time.

45. Before you begin, prepare for the dissection:
  a. Prepare a 7 mL universal tube per sample with 2 mL (adult brain) or 1 mL (neonatal or embryonic brains) papain dissociation medium.
    i. Add L-cysteine to the papain dissociation medium (see materials and equipment) and incubate for 10 min at 37°C.
46. Humanely sacrifice an animal in accordance with local regulations.
47. Dissect the brain (as described in Preparing acute brain slices) and place the whole brain, or the dissected region of interest in a large weighing boat.
48. Quickly cut up the brain in small pieces using a scalpel.
49. Add the tissue pieces to papain dissociation medium.
  a. Pool 2–3 embryonic or neonatal brains in 1 mL papain dissociation medium.
  b. For adult samples, dissociate in 2 mL papain dissociation medium.
50. Use scissors to cut the tissue into smaller pieces.
51. Incubate for 1 h at 37°C.
52. During the incubation, prepare for the myelin removal:
  a. Cool the ovomuccoid, PBS and BSA on ice.
  b. Prepare 7 × 15 mL tubes (if using 3 columns per sample) per sample and 1 × 50 mL tube per sample.
  c. Prepare the LS MACS columns, MidiMACS Separators, and MACS Multistand.
53. Inhibit digestion with 1 mL ovomuccoid.
54. Resuspend the tissue and transfer to a 15 mL tube.
55. Complete to 8 mL with ovomuccoid.
56. Centrifuge for 10 min at 300 *g*.
57. Wash in 7 mL ice-cold PBS.
58. Proceed with the myelin removal using Myltenyi Biotec Myelin Removal Beads II following the manufacturer’s instructions for magnetic labeling and depletion with LS columns: https://www.miltenyibiotec.com/GB-en/products/myelin-removal-beads-ii-human-mouse-rat.html#gref Troubleshooting 7.
  a. We recommend filtering the samples before adding the Myelin Removal Beads. For best results, use a 70 μm cell strainer (as an additional step between steps 2 and 3 of the magnetic labeling in the Myltenyi protocol).
  b. Ensure that you wait for the columns to be empty before applying the wash (step 4 of the depletion with LS columns in the Myltenyi protocol).
  c. At the end, combine the flow-through from all columns used for the same sample.

**CRITICAL:** Perform the myelin removal on ice, and in a microbiological safety cabinet to preserve RNA.

**Figure 5:**
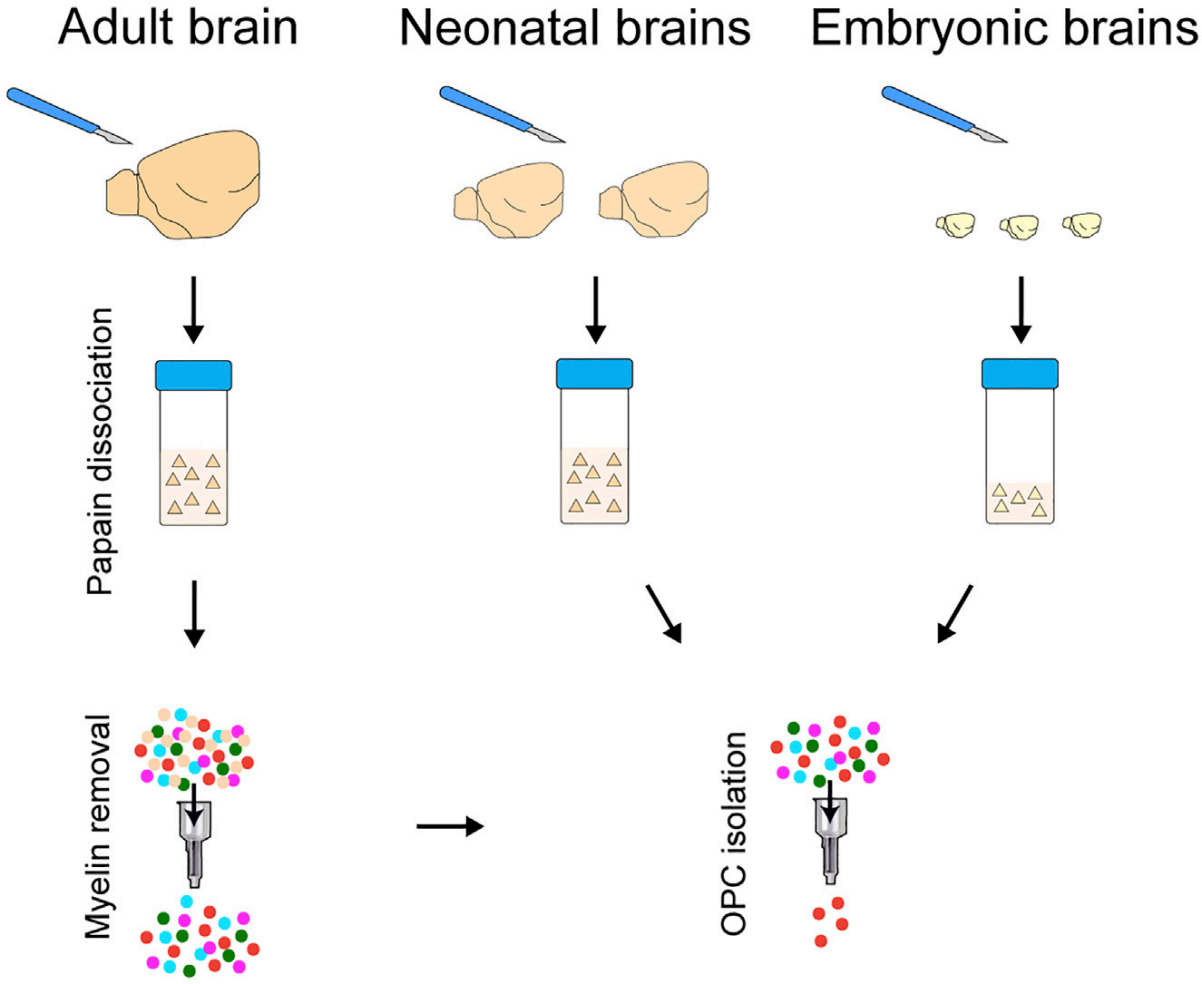
MACS isolation of OPCs Diagram showing the pipeline to isolate OPCs from adult, neonatal, and embryonic brains using Magnetic Associated Cell Sorting (MACS).

***Optional:*** For embryonic and neonatal samples, omit the myelin removal step, as it is not necessary, and may cause tissue loss (Figure 5).

59. Proceed with the OPC isolation using Myltenyi Biotec CD140 MicroBeads following the manufacturer’s instructions for OPC isolation: https://www.miltenyibiotec.com/GB-en/products/cd140a-pdgfra-microbead-kit-mouse.html?countryRedirected=1#gref Troubleshooting 7.
  a. Be sure to follow the instructions for magnetic labeling and magnetic separation with MS columns (rather than depletion).
  b. During the first centrifugation step (step 2 of the magnetic labeling in the Myltenyi protocol), prepare the MS columns, the MiniMACS Separators, and the MACS MultiStand, as well as 2 × 15 mL tubes per sample.
  c. Be sure to remove the columns from the MiniMACS Separators before plunging them to collect the isolated OPCs.

**CRITICAL:** Perform the OPC sort on ice, and in a microbiological safety cabinet to preserve RNA.

**Figure 6:**
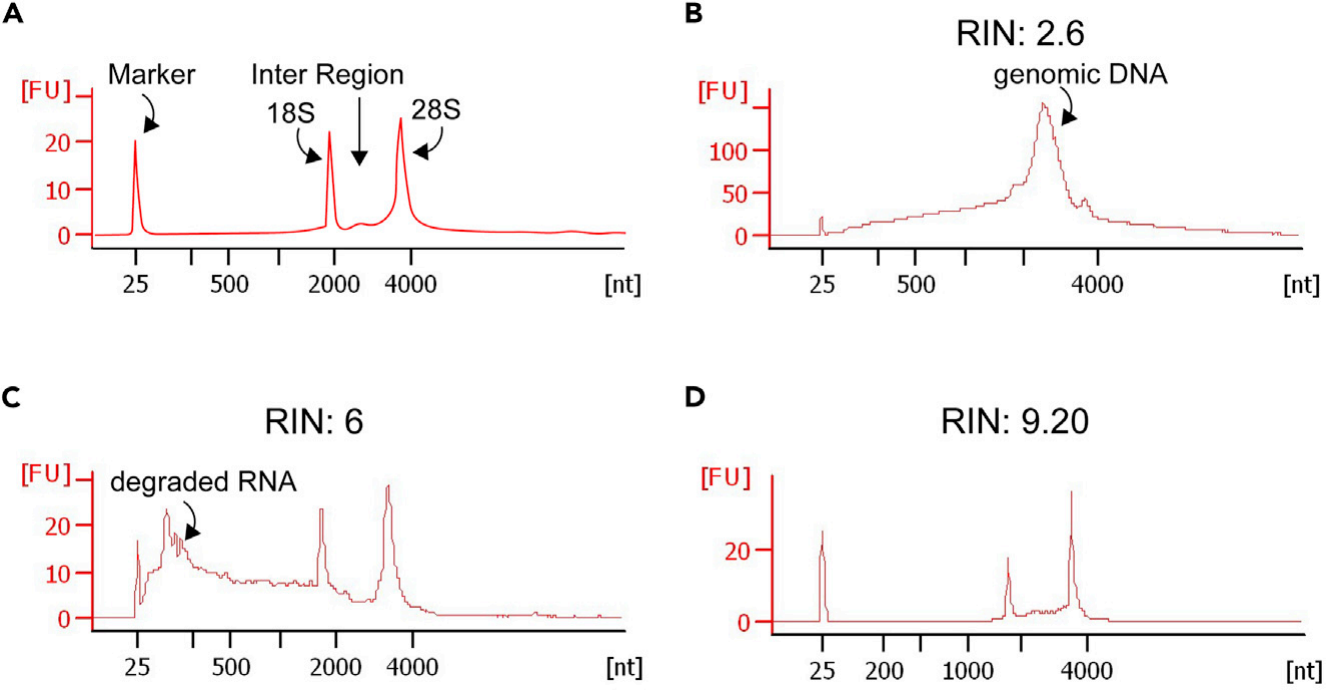
Representative bioanalyzer electrophoregrams of RNA samples (A) Schematic of an electrophoregram of an RNA sample with a RIN value of 10. There are clear peaks for a marker and ribosomal RNA (18S and 28S). (B) Sample with high levels of genomic DNA contamination, as evidenced by the high signal in the inter region between the 18S and 28S peaks. This occurs for example when omitting DNase during the RNA extraction (see step-by step method details step 60a). (C) Sample showing RNA degradation (with high signal immediately after the marker peak), and therefore, a low RIN. The samples shown in B and C should not be sequenced. (D) Sample with good quality RNA and a high RIN value.
  b. Be sure to add β-mercaptoethanol to buffer RLT, as cells isolated from tissues will be rich in RNAses (as described in the first note of the Qiagen Protocol; see Figure 6C).

60. Proceed with RNA extraction using Qiagen’s RNeasy Micro Kit according to the manufacturer’s instructions: https://www.qiagen.com/gb/products/discovery-and-translational-research/dna-rna-purification/rna-purification/total-rna/rneasy-micro-kit/?clear=true#orderinginformation
  a. Be sure to include DNase when performing step 5 in the Qiagen protocol. Failure to do so leads to genomic DNA contamination (Figure 6B).

**CRITICAL:** β-mercaptoethanol is acutely toxic. Wear gloves and manipulate in a fume hood when adding to buffer RLT.

**CRITICAL:** Perform the RNA extraction in a microbiological safety cabinet, to preserve RNA.

**Pause point:** RNA can be kept at −80°C until all the samples have been processed.

61. Run a quality control check on the RNA
  a. Quantify the RNA in your samples, using a Qubit Fluorometer as this will determine which kit is needed to prepare the cDNA library. For instance, after MACS sorting OPCs, we found that our RNA concentration fell in the 250 pg–10 ng range, and therefore used the kit described in step 62.
  b. Measure the RNA Integrity Number (RIN; see Schroeder et al., 2006) using an Agilent Bioanalyzer. Typically, RIN values above 7 can be used for analysis (Sheng et al., 2017) (Figure 6).
62. Prepare the RNA-seq library using Takara Clontech’s SMARTer Stranded Total RNA-seq Kit v2 - Pico Input Mammalian according to the manufacturer’s instructions: https://www.takarabio.com/learning-centers/next-generation-sequencing/technical-notes/rna-seq/stranded-libraries-from-picogram-input-total-rna-(v2)
63. Sequence the libraries on an Illumina sequencer (or another appropriate instrument).
64. Assess read quality and trim the reads using standard protocols (for instance, *Trim Galore!* (https://www.bioinformatics.babraham.ac.uk/projects/trim_galore).
65. Align the reads to a reference mouse genome (such as e GRCm38/mm10) using standard protocols (for instance, *Tophat* (version 2.1.1, https://ccb.jhu.edu/software/tophat/index.shtml).
66. Proceed with normalization and differential expression analysis with appropriate software and protocols (for instance, the R Bioconductor DESeq2 package (Love et al., 2014; https://bioconductor.org/packages/release/bioc/html/DESeq2.html).
67. Perform any other required analyses, such as GO term analysis with appropriate software and protocols (for instance DAVID (https://david.ncifcrf.gov/) and REVIGO (Supek et al., 2011; http://revigo.irb.hr)).

***Note:*** Unless you have a strong background in bioinformatics, we recommend collaborating with a bioinformatician to analyse RNA sequencing data.

## Expected outcomes

**Figure 7:**
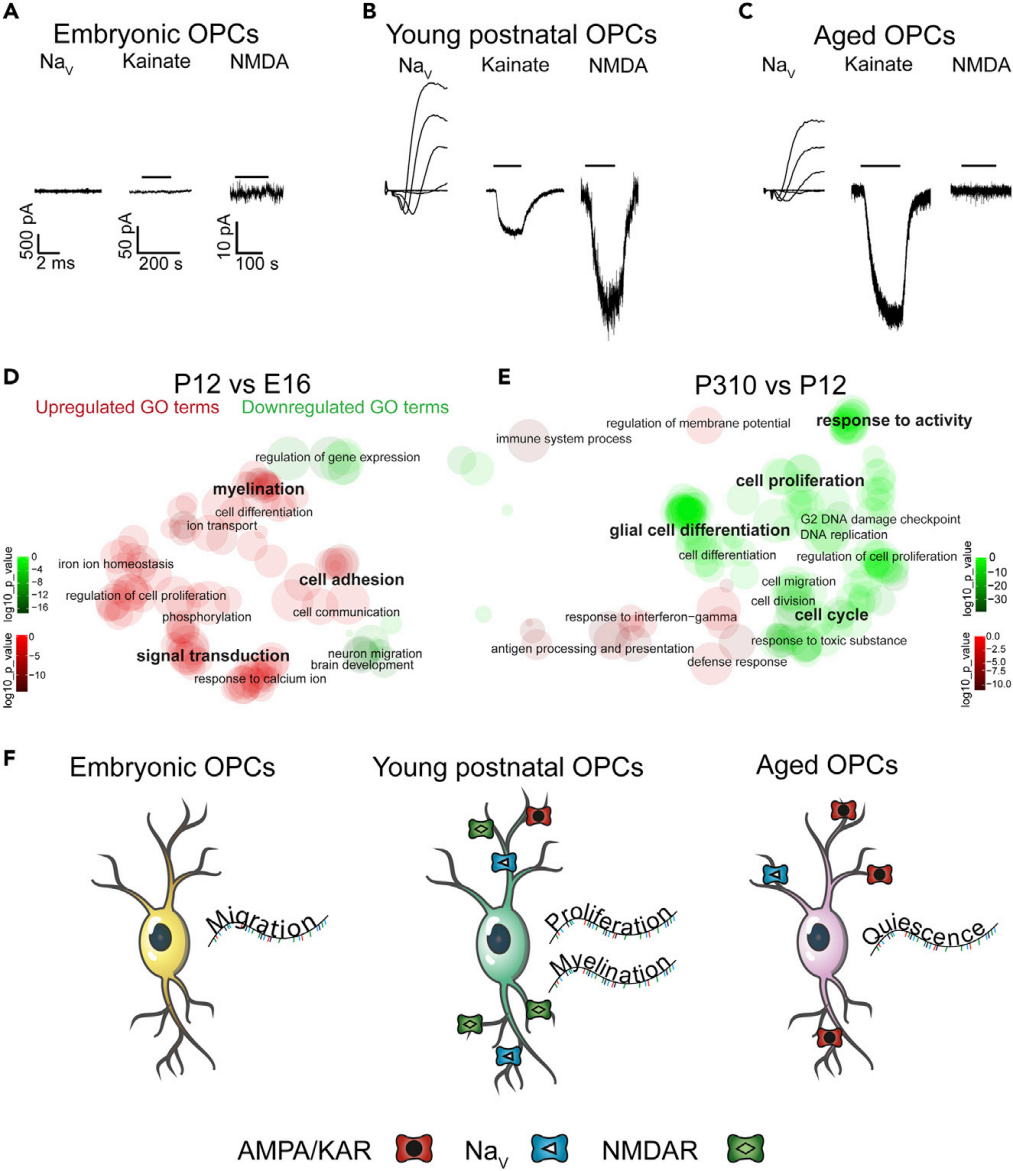
Whole-cell voltage clamp and bulk RNA sequencing of embryonic, young postnatal, and aged OPCs Panels A–C show representative leak-subtracted voltage-gated Na^+^ channel (Na_V_) currents and responses to 30 μm kainate and 60 μm NMDA application in (A) embryonic slices, (B) neonatal slices, and (C) aged slices. Panels A-C are on the same scale. Panels D and E show GO analysis of gene transcripts in OPCs with age. Altered GO terms between (D) young postnatal (P12) and embryonic (E16) OPCs or (E) aged (P310) and young postnatal OPCs are displayed. (F) summarizes the correlated electrophysiological and differential RNA expression associated with embryonic, young postnatal and aged OPCs. Panels A-E are adapted from Spitzer et al., 2019 (with permission according to a Creative Commons Attribution License (CC BY)).

With this protocol, it is possible to determine passive membrane properties, and voltage-gated ion channel and neurotransmitter receptor expression in cells in acute brain slices at different ages (Figures 7A–7C), and in different brain regions (Spitzer et al., 2019). In addition, dye loading during whole-cell patch clamp allows for morphological characterization, cell location determination, and investigation of protein expression (Figure 5).When cells are physiologically homogeneous within a condition (such as within a timepoint or brain region), bulk RNA sequencing provides additional information on cell function (Figures 7D and 7E). Together, these data can determine cell identity or state and cell function (Figure 7F; Spitzer et al., 2019).

## Quantification and statistical analysis

### Calculating R_s_, C_m_, and R_m_

**Figure 8:**
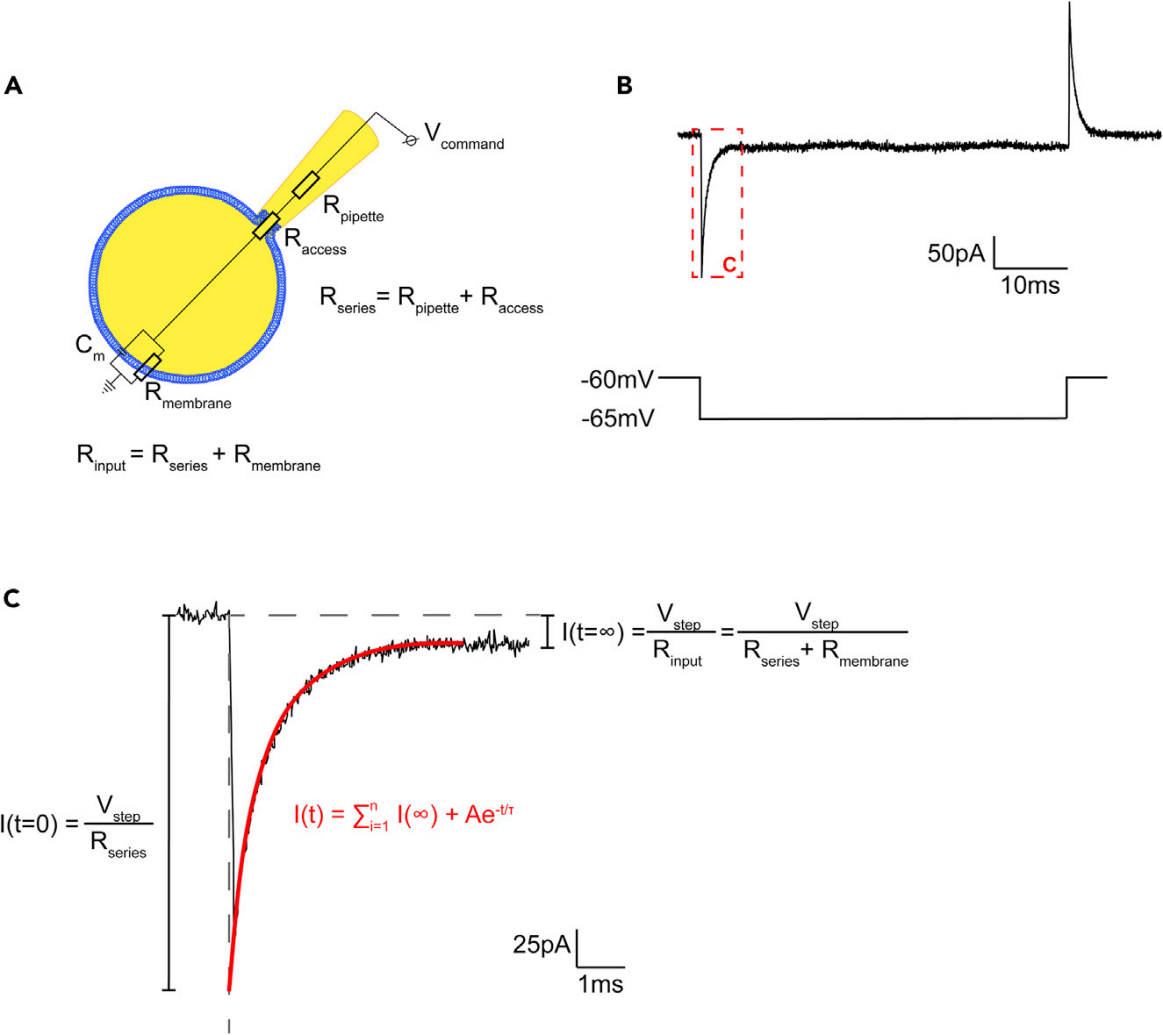
Analyzing Rs, Cm and Rm from capacitive transients (A) Diagram of the electrical circuit when recording from OPCs in whole-cell voltage clamp mode. (B) Representative current response (top) elicited by a −5 mV voltage step (bottom), in voltage clamp mode. When R_s_ is uncompensated, this current response can be used to measure R_s_, C_m_ and R_m_. (C) At the onset of the voltage pulse, t=0, the current only flows through R_s_, allowing us to calculate it using Ohm’s law. However, the current at t=0 is not recorded due to pipette capacitance; thus, we fit the current response to a single exponential (in red), to extrapolate the current at the onset of the voltage pulse. Once the current has reached a steady state, we can calculate R_m_.

Here we describe how to measure series resistance (R_s_) and passive membrane properties including cell capacitance (C_m_) and membrane resistance (R_m_) from capacitive transients elicited by a −5 mV pulse (Figure 8B). In whole-cell voltage clamp of a compact cell, the electrical circuit formed comprises a membrane capacitance, a membrane resistance, and the series resistance (comprised of the pipette resistance and the access resistance), in series with the membrane capacitance and resistance (Figure 8A).

1. To measure R_s_, we take advantage of the fact that when a voltage step (V_step_) is initiated (t=0), the charge across the membrane capacitor and resistor is null (Figure 8C). Thus, the current flow I, is

$$I_{t0}=\frac{{\text{V}}_{step}}{R_{s}}$$

and R_s_ is

$$R_{s}=\frac{{\text{V}}_{step}}{I_{t0}}$$

2. To measure R_m_, we take advantage of the fact that at steady state (t=∞), the membrane capacitor is fully charged and current only flows through the resistors. Thus, the current flow I, is

$$I_{t\infty }=\frac{{\text{V}}_{step}}{R_{s}+R_{m}}$$

Isolating R_m_ and substituting R_s_ from the equation above, we find

$$R_{m}={\text{V}}_{step}\cdot \frac{I_{t0}-I_{t\infty }}{I_{t\infty }\cdot I_{t0}}$$

3. To find $I_{t0}$ and $I_{t\infty }$, we use the fact that the current flow through this circuit can be described by the following single exponential (Tessier-Lavigne et al., 1988):

$$I\left(t\right)=\frac{{\text{V}}_{step}}{R_{s}+R_{m}}\;\cdot \left(1+\frac{R_{m}\cdot e^{t/\tau }}{R_{s}}\right)$$

Thus, we fit the recorded capacitive transient using a single exponential,

$$I\left(t\right)=\overset{n}{\underset{i=1}{\sum }}I\left(\infty \right)+A\cdot e^{-t/\tau }$$

and extrapolate the curve to the onset of the voltage pulse (Figure 8C), allowing us to determine $I_{t0}$ and $I_{t\infty }$, and therefore to calculate R_s_ and R_m_.

4. From this single exponential fit, we also determine τ, the time constant of current decay. τ is also described as

$$\tau =C_{m}\cdot \frac{R_{s}\cdot R_{m}}{R_{s}+R_{m}}$$

5. Rearranging this equation, we can calculate C_m_:

$$C_{m}=\tau \cdot \frac{R_{s}+R_{m}}{R_{s}\cdot R_{m}}$$

We perform this fitting and calculate R_s_, R_m_ and C_m_ with a lab written Matlab script (available upon request), using the equations described above.

### Measuring the peak voltage-gated Na^+^ current

Here we describe how to measure current responses from the activation of voltage-gated Na^+^ channels during a voltage-step protocol. We use a lab written Matlab script (available upon request) to perform the steps described below.

6. Subtract the capacitive and leak currents.
  a. We perform this subtraction mathematically. To do so, we assume that the capacitive and leak currents follow Ohm's law, and are proportional to the amplitude of the voltage pulse applied. We use the current resulting from the −100 mV pulse as our template leak current, as there are no voltage-gated transients at this potential during the time frame in which voltage-gated Na^+^ channels activate, and scale it to each voltage pulse using a multiplier, mul:
    $$mul_{i}=\frac{-\left(V_{step\left(i\right)}-V_{hold}\right)}{-100-V_{hold}}$$
    such that, for a holding potential $V_{hold}$ of −60 mV (uncorrected for junction potential),

**Table:** 

Voltage step, $V_{step\left(i\right)}$	Multiplier, $mul_{i}$
−120 mV	−1.5
−100 mV	−1
−80 mV	−0.5
−60 mV	0
−40 mV	0.5
−20 mV	1
0 mV	1.5
20 mV	2
40 mV	2.5
  b. We then subtract these scaled currents from our recorded currents, using the following equation:
    $$I_{sub\left(i\right)}=\;I_{i}+mul_{i}\cdot \left(I_{100}-I_{baseline}\right)-I_{baseline}$$
    and obtain capacitance and leak subtracted current responses (Figure 9).

**Figure 9:**
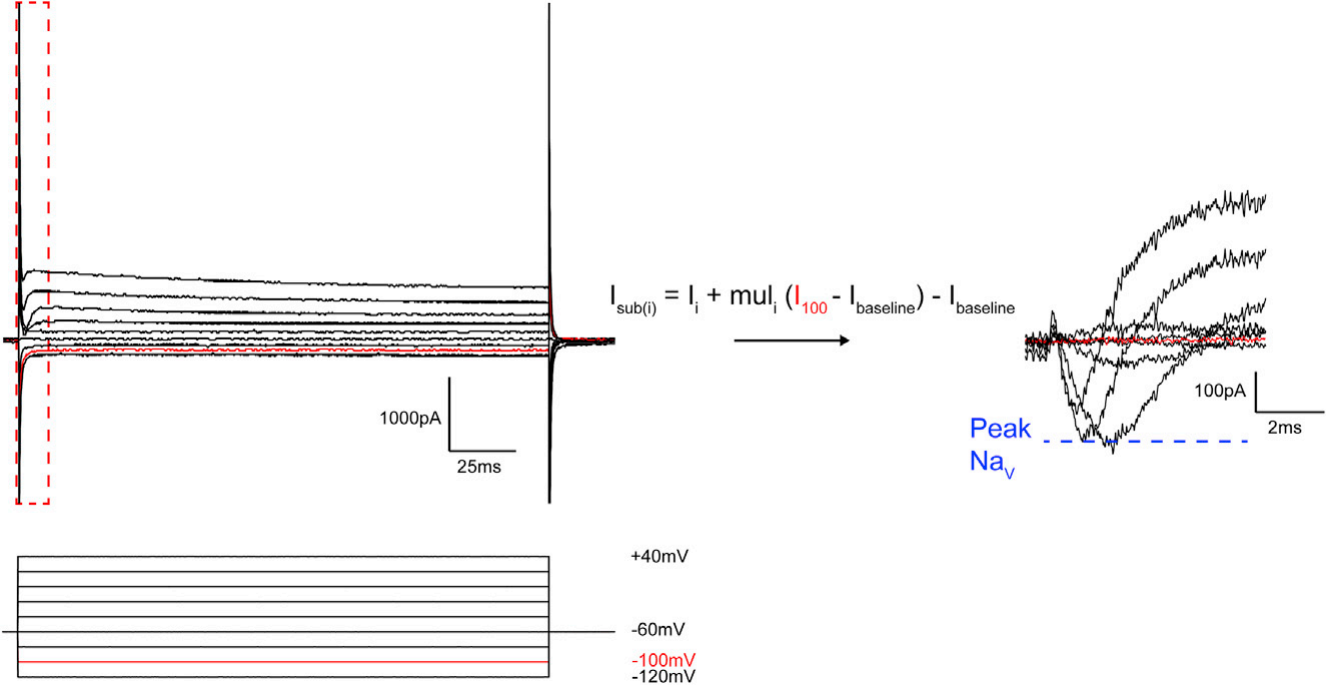
Analyzing voltage-gated Na^+^ channel currents We measure voltage-gated Na^+^ channel currents by recording the current response to a voltage step protocol ranging from −120 mV to +40 mV (not corrected for junction potential). The left panel shows the voltage steps and a representative current response. To isolate Na^+^ currents, we first perform leak and capacitive current subtraction using the current at the −100 mV step (in red) as our template leak current and scaling it to the other voltage steps. From the subtracted current responses (right panel), we measure the peak Na^+^ current as the peak inward current (blue line) during the first 7 ms of the voltage steps.

7. From these subtracted currents, we measure the peak voltage-gated Na^+^ channel current as the peak inward current in the first 7 ms following the onset of the voltage pulse (Figure 9).

### Measuring NMDA and kainate current responses

**Figure 10:**
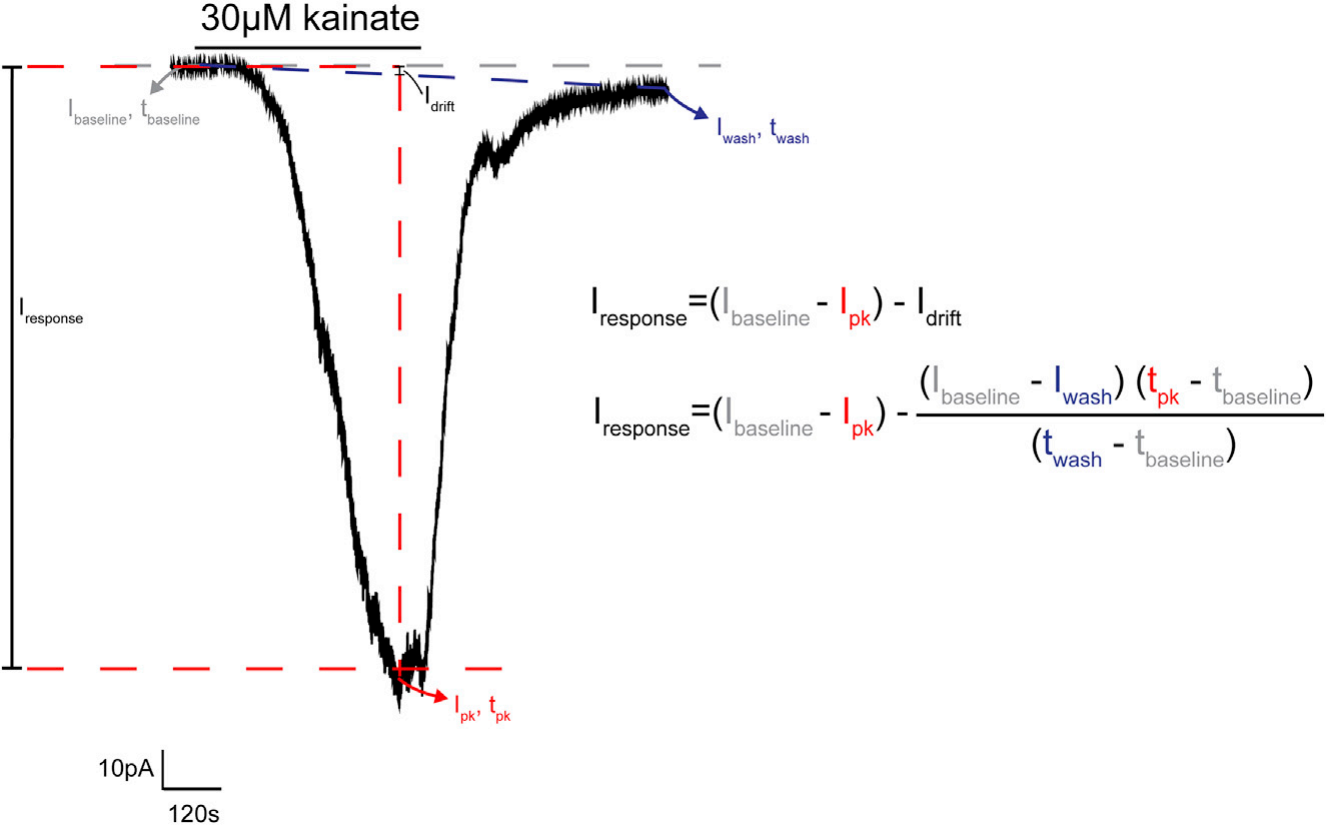
Analyzing kainate or NMDA receptor currents Representative response to the bath application of 30 μm kainate. To measure the peak response to kainate (or NMDA), we subtract the baseline current (I_baseline_) from the peak current (I_peak_). However, we also account for a baseline drift (I_drift_), as this can occur with during long drug applications.

Here we describe how to measure current responses to NMDA and kainate application (Figure 10).

8. Open the recording in Clampfit.
9. Measure the baseline current, I_baseline_, at t_baseline_.

***Note:*** For noisy recordings, we suggest filtering the recording before measuring current responses using a lowpass Bessel filter. This is particularly useful for NMDA-induced responses, as they are typically small (2–10 pA), and can fall within the noise level in unfiltered recordings.

10. Measure the peak current, I_pk_, at t_pk_.
11. Measure the recovery current, I_wash_, at t_wash_. This is measured once the current has recovered to a stable baseline (Figure 10).
12. To account for the potential baseline drift during the drug application, calculate the current response, as (Figure 10):
$I_{response}=\left(I_{baseline}-I_{pk}\right)-I_{drift}$

where

$$\frac{I_{drift}}{\left(I_{baseline}-I_{wash}\right)}=\frac{\left(t_{pk}-t_{baseline}\right)}{\left(t_{wash}-t_{baseline}\right)}$$

rearranging and substituting $I_{drift}$ in the first equation, we find

$$I_{response}=\left(I_{baseline}-I_{pk}\right)-\frac{\left(I_{baseline}-I_{wash}\right)\cdot \left(t_{pk}-t_{baseline}\right)}{\left(t_{wash}-t_{baseline}\right)}$$

13. To calculate receptor density, divide the current response by the membrane capacitance, which is a proxy for cell size.

***Optional:*** If all cells in each condition have the same capacitance, the total current can be compared.

## Limitations

We described a protocol to measure ion channel currents and neurotransmitter receptor currents in cells from different timepoints and/or brain regions. However, the external and internal solution recipes we describe here are specific to our purposes, and different components may be needed for other purposes. For instance, we use BAPTA as a Ca^2+^ chelator in our internal solution, as we found that it helped with stability and response rundown during long recordings; however, if interested in G-protein coupled receptors, you may wish to use EGTA, a less potent chelator.

Here, we describe a whole-cell patch clamp protocol, and not perforated patch. When interested in downstream intracellular signaling, using perforated patch may be more suitable, as the native intracellular milieu is more conserved than in whole-cell patch clamp, where it is mostly replaced by the internal solution.

We perform our experiments at room temperature (19°C–23°C), and in HEPES-buffered aCSF, rather than physiological temperature. Thus, receptor kinetics are difficult to interpret. Recording in bicarbonate-buffered aCSF at physiological temperature would be more appropriate if focusing on receptor kinetics, but the perfusion flow must be constant so that pH is maintained with 95% O_2_ / 5% CO_2_ bubbling. In addition, we typically bath apply receptor agonists such as NMDA and kainate. As bath application requires some time for the whole bath to reach concentration, it should not be used when attempting to study receptor kinetics, as receptor desensitization does occur. Following this, the flow rate should be constant between experiments. Alternatives to bath application include fast perfusion systems, where a pipette placed in the bath near the cell is used for drug delivery, or puffer systems, where a pulse of positive pressure is used to eject a small volume of drug onto a cell.

Cell recovery after dye loading can be challenging. With practice, approximately 60% of cells remain in the slice following a recording; however, this proportion decreases with longer recordings. In addition, not all recovered cells can be found when imaging a post-hoc staining. Thus, while dye loading provides morphology, location, and protein expression data, it is difficult to obtain these data for every recorded cell.

While electrophysiology remains the preferred method to study voltage-gated ion channel and neurotransmitter receptor function, it can be difficult to link physiological properties to cell function. Combining multiple approaches, such as electrophysiology, cell morphology, immunohistochemistry, and bulk RNA sequencing, as we have described above, can be useful to assess whether cell subtypes, or states, characterized by a specific electrophysiological profile are more likely to perform specific functions. However, bulk RNA sequencing is only appropriate for this purpose when cells are relatively homogeneous within a condition. If the cells display heterogeneity with respect to their physiological properties, patch-seq (single-cell sequencing of RNA extracted from a patched-cell) is likely to be more appropriate, although bulk sequencing can be complementary to patch-seq in this case.

Finally, MACS may not be an appropriate sorting method for all cell types. For instance, microglia are activated by MACS sorting, and thus, for these cells, Fluorescence Associated Cell Sorting (FACS) is more appropriate. We refer the reader to the following protocol for FACS (Barbar et al., 2020).

## Troubleshooting

### Problem 1: The acute brain slices are unhealthy

Most of the neurons on the brain slice are dead or dying (steps 4 and 5).

### Potential solution

The dissection may not have been fast enough. Try to increase your dissection speed. Ensure that you do not damage the brain extensively while dissecting and slicing, as this may increase cell death.

Check your slicing and resting aCSF and make sure that you have included glucose in the solution (a sign of glucose omission is that blood vessels in the slice become very prominent). Measure the osmolarity of the slicing and resting aCSF, which is expected to be 315–330 mOsm.

### Problem 2: Difficulty to seal cells

The resistance increases when you begin applying negative pressure, but quickly decreases again, or you suck up the cell (step 22).

### Potential solution

Try pulling pipettes with a slightly higher resistance. OPCs are very small cells, and it can be challenging to start patching them with lower resistance pipettes. When training to patch OPCs, start with pipettes around 7 MΩ, and make your way down to 5–6 MΩ.

### Problem 3: Difficulty to seal cells

The cell does not seal when you apply negative pressure (step 22).

### Potential solution

If this occurs occasionally, you may have selected an unhealthy cell. Try selecting less shiny or grainy cells.

If this occurs systematically, there may be a leak between the 1 mL syringe where you apply pressure and the electrode holder. Check that you can apply positive pressure; if this doesn’t work, check for cracks in the syringe, valve, tubing or holder to identify a potential leak, and replace the faulty component.

If you can still apply positive pressure, a leak is unlikely to be the answer. Check slice health. If your slice is unhealthy, discard it and change to another slice.

If your slice is healthy, your solutions may be the problem. Try patching the cells with another internal solution if you have one available.

If your internal solution is fine (changing internal does not help with patching), check the osmolarity of your external solutions.

If the cell is taking a long time to seal, make yourself a cup of tea and it may seal in the meantime.

### Problem 4: Difficulty opening the cell

The cell does not open once you have achieved a giga-ohm seal (step 24).

### Potential solution

Try pulling pipettes with a slightly lower resistance.

Too much negative pressure applied during sealing, apply less (or try stopping applying positive pressure when close to the cell and allow the cell to seal itself).

### Problem 5: Difficulty opening the cell

The seal is lost as you open the cell (step 24).

### Potential solution

Try using less negative pressure to open the cell.

If this happens systematically, it may indicate a problem with slice health, or your solutions. Check slice health. If your slice is unhealthy, discard it and change to another slice.

If your slice is healthy, your solutions may be the problem. Try patching the cells with another internal solution if you have one available.

If your internal solution is fine (changing internal does not help with patching), check the osmolarity of your external solutions.

### Problem 6: The flow through the gravity-fed perfusion stops

The flow rate is significantly decreased or stops (step 28).

### Potential solution

There may be bubbles in the line. Make sure that you prime the lines before you start your patching day (run the solution through until bubbles are gone). Clear any bubbles that appear during the day.

There may be bacteria growing in the lines, as all solutions flowing through the lines contain glucose. Flush the lines with bleach and wash with distilled water.

### Problem 7: MACS columns are blocked during myelin retrieval or OPC sort

The tissue suspension does not flow through the columns (steps 58 and 59).

### Potential solution

Add DNase (0.04 mg/ml) to the 0.5% BSA MACS buffer.

## Resource availability

### Lead contact

Further information and requests for resources and reagents should be directed to and will be fulfilled by the lead contact, Ragnhildur Thóra Káradóttir (rk385@cam.ac.uk).

### Materials availability

This study did not generate new reagents.

### Data and code availability

This study did not generate new data nor code, but Matlab scripts to measure R_s_, C_m_ and R_m_ and voltage-gated Na^+^ currents are available upon request.
